# In vitro gentamicin exposure alters caveolae protein profile in cochlear spiral ligament pericytes

**DOI:** 10.1186/s12953-018-0132-x

**Published:** 2018-03-16

**Authors:** Elisa Ghelfi, Yohann Grondin, Emil J. Millet, Adam Bartos, Magda Bortoni, Clara Oliveira Gomes dos Santos, Humberto J. Trevino-Villarreal, Rosalinda Sepulveda, Rick Rogers

**Affiliations:** 1 Harvard T.H. Chan School of Public Health, Department of Environmental Health, MIPS Program, Boston, MA USA; 2 Universidade de Sao Paulo, Faculdade de Medicina, Sao Paulo, Brazil; 3 Harvard T.H. Chan School of Public Health, Department of Genetics and Complex Diseases, Boston, MA USA; 4 Universidad Autónoma de Nuevo León, Facultad de Medicina, Monterrey, Mexico

**Keywords:** Ototoxic drug, Nonsyndromic hearing loss, Rab GTPase, GOrilla enrichment analysis, Proteomaps

## Abstract

**Background:**

The aminoglycoside antibiotic gentamicin is an ototoxic drug and has been used experimentally to investigate cochlear damage induced by noise.

We have investigated the changes in the protein profile associated with caveolae in gentamicin treated and untreated spiral ligament (SL) pericytes, specialized cells in the blood labyrinth barrier of the inner ear microvasculature. Pericytes from various microvascular beds express caveolae, protein and cholesterol rich microdomains, which can undergo endocytosis and transcytosis to transport small molecules in and out the cells. A different protein profile in transport-specialized caveolae may induce pathological changes affecting the integrity of the blood labyrinth barrier and ultimately contributing to hearing loss.

**Method:**

Caveolae isolation from treated and untreated cells is achieved through ultracentrifugation of the lysates in discontinuous gradients. Mass spectrometry (LC-MS/MS) analysis identifies the proteins in the two groups. Proteins segregating with caveolae isolated from untreated SL pericytes are then compared to caveolae isolated from SL pericytes treated with the gentamicin for 24 h. Data are analyzed using bioinformatic tools.

**Results:**

The caveolae proteome in gentamicin treated cells shows that 40% of total proteins are uniquely associated with caveolae during the treatment, and 15% of the proteins normally associated with caveolae in untreated cell are suppressed. Bioinformatic analysis of the data shows a decreased expression of proteins involved in genetic information processing, and an increase in proteins involved in metabolism, vesicular transport and signal transduction in gentamicin treated cells. Several Rab GTPases proteins, ubiquitous transporters, uniquely segregate with caveolae and are significantly enriched in gentamicin treated cells.

**Conclusion:**

We report that gentamicin exposure modifies protein profile of caveolae from SL pericytes. We identified a pool of proteins which are uniquely segregating with caveolae during the treatment, mainly participating in metabolic and biosynthetic pathways, in transport pathways and in genetic information processing. Finally, we show for the first time proteins associated with caveolae SL pericytes linked to nonsyndromic hearing loss.

**Electronic supplementary material:**

The online version of this article (10.1186/s12953-018-0132-x) contains supplementary material, which is available to authorized users.

## Background

The aminoglycoside antibiotic gentamicin (GTM) used in the treatment of bacterial infections has important ototoxic side effects. It can induce vestibulotoxicity characterized by vertigo and dizziness and cochleotoxicity, manifesting as tinnitus and permanent sensorineural hearing impairment and loss [1]. The incidence of hearing loss is up to 25% among patients treated with the antibiotic [2]. GTM is used to treat infections in a wide demographic span from adults, to children, to infants including preterm infants. In children and neonates the incidence of cochlear and vestibular toxicity is less than in adults [3]. The risk of ototoxicity increases when GTM is administered concurrently with other ototoxic drugs such as chemotherapeutic agents, loop diuretics or glycopeptides antibiotics [4, 5] and/or concurrently with noise exposure [3] old age [1] and in patients with mitochondrial DNA mutation 1555A > G. [1, 5, 6].

**Fig. 1:**
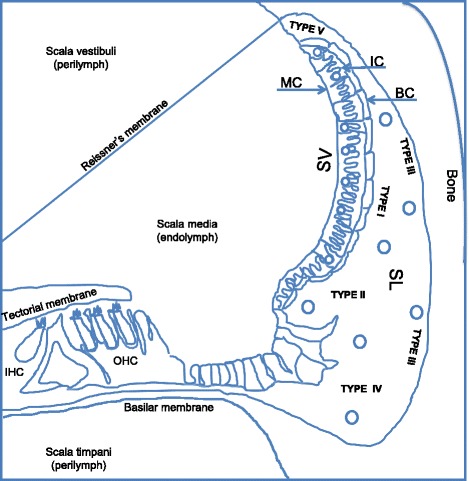
Cochlea and lateral wall schematic. The cochlea is part of the inner ear; it is the organ of hearing. It harbors at its center the membranous labyrinth where the sensory cells reside. The cochlea is a spiral shell-like structure; it is encased in the temporal bone and contains three canals spiraling in two and one half turns. Two of the canals, scala tympani and scala vestibuli are filled with perilymph a fluid similar to the cerebrospinal fluid and plasma ultrafiltrate [116]. A third canal named scala media is separated from scala tympani and scala vestibuli by two membranes rich in tight junctions, the Reissner’s membrane and the Basilar membrane respectively. Scala media contains the endolymph a uniquely potassium-rich, positively polarized fluid, originating from the active filtration of the SV. The SV and the SL form the lateral wall of the inner ear, their microvasculature constitutes the blood labyrinth barrier (BLB) and functions with the tissue highly specialized cells to maintain the ionic composition of the endolymph and perilymph. Three different cell types are recognized in the SV; marginal, intermediate and basal cells. The marginal cells (MC) secrete K^+^, they constitute a homogeneous layer of epithelial cells lining the scala media fluid space, connected by tight junctions, adherens junctions and desmosomes. Marginal cells are rich in microvilli on the luminal side and lack a basement membrane on the opposite side, directly associating them with the vasculature beneath them [117]. Intermediate cells (IC) rich in melanin granules intertwine with the marginal cells without reaching the luminal side. Basal cells (BC) are lateral to the intermediate cell layer adjacent to the SL. The SL comprises five types of fibrocytes (I-V). The fibrocytes participate in pumping K^+^ out of the perilymph (Type II, IV, and V) and transport it to generate the endochoclear potential in the endolynph (Type I) [117]. In the figure: stria vascularis (SV). Spiral ligament (SL), marginal cells (MC), intermediate cells (IC), basal cells (BC), inner hair cells (IHC), outer hair cells (OHC), fibrocytes type I-V (Type I-V), circles are schematic representation of microvessels

Despite its toxic side effects GTM is one of the antibiotics of choice worldwide due to the relatively low cost and its wide spectrum of action comprising many strains of Gram-positive and Gram-negative pathogens [4]. GTM can be administered intravenously or intramuscularly. Ototoxicity correlates with plasma concentration and duration of the therapy [7]. The symptoms can arise during the drug administration or after the end of the therapy; hence GTM is usually delivered in an inpatient setting when the drug blood level can be routinely monitored. GTM penetrates all cell types of the cochlea, and continues to accumulate in the inner ear even after the termination of its administration [8–11]. Following systemic administration, GTM and the aminoglycosides are presumably trafficked through the endothelial cells in the microvasculature of the cochlear lateral wall into the perilymphatic fluids [3]. More recent evidences strongly suggest that aminoglycosides may be predominantly trafficked through the blood labyrinth barrier (BLB) into the SV and into the endolymph before entering the cochlear hair cells. Within the inner ear perilymph, plasma concentrations of GTM increase in a time and dose dependent manner. The recognized cellular basis for aminoglycoside-induced hearing loss is the death of mechanosensory cochlear hair cells. Chronic GTM treatment leads to cell loss and reduction of thickness of the stria vascularis (SV) [12]. In guinea pigs a single dose of GTM, systemically or locally administered, persists in SL fibrocytes, suggesting that hair cell vulnerability may be influenced by the state of spiral ligament cells [11]. Although the molecular mechanisms are poorly understood, aminoglycoside antibiotics generate free radicals within the inner ear, damaging sensory cells and neurons and inducing apoptosis [13]. Two major vascular beds form the lateral wall microvasculature of the inner ear, the SV and the SL, deliver about 80% of the blood supply to the cochlea. The SL microvasculature surrounding the SV controls the blood flow directed to the stria vascular bed [14, 15] through contractile proteins of the SL pericytes (Fig. 1). Pericytes also control other important functions in the microvasculature physiology such as: maintenance of the blood-labyrinth barrier (BLB), signaling pathways to endothelial cells, and modulation of the microvessel wall permeability [8]. Transport of macromolecules across the BLB via transcytotic and endocytotic mechanisms constitute another likely function of the pericytes in the inner ear. Recently, it has been shown that, in the blood brain barrier (BBB), pericyte signaling to endothelial cells control the vesicular transcytosis through the up- or down-regulation of Mfsd2a protein on the cell surface of endothelial cell membrane [16]. Caveolae are cholesterol rich membrane microdomains found on many cell types and particularly abundant on endothelial cells and adipocytes. Pericytes from various microvascular beds have also been described expressing caveolae, transporting small molecules into the cells and unloading cargoes into the extracellular space [17]. Caveolae are described mainly on the cell surface and in the cytoplasm; they are constituted of cholesterol, phospholipids, sphingolipids and proteins. Caveolae contain constitutive proteins such as caveolin 1 (cav1), caveolin 2 (cav2) and caveolin 3 (cav3). Cav1 is a structural protein essential for caveolae formation observed also in the nucleus [18] in the cytoplasm, and in organelles such as mitochondria [19]. Cav1 and cav2 are abundant in non-muscle cells, Cav3 is found in skeletal muscles and in some smooth-muscle cells. Ablation of cav1 or cav3 but not cav2 causes disassembly and loss of caveolae [20].

Several members of the Rab GTPase family have been shown to coordinate and participate in caveolae endocytosis [21, 22]. Proteins of the Rab GTPase family are cellular regulators of vesicular transport and membrane trafficking. In humans, the recognized members of the Rab family account for more than 60 proteins localized in various cellular membranes. Rabs alternate between the active GTP (guanosine triphosphate)-bound and the inactive GDP (guanosine diphosphate)-bound conformation, and participate in specifying transport pathways in the intracellular membrane trafficking system of all eukaryotes. These pathways comprise endocytosis, exocytosis, phagocytosis, recruitment of tethering factors, control of anterograde and retrograde trafficking between membranes and organelles, and the coordination of cargo delivery and membrane recycling [23, 24]. Finally, proteins relevant for hearing loss have been shown to segregate with caveolae. In particular, in their analysis of proteins associated with cholesterol-rich membrane microdomains in the inner ear cells, Thomas P. and colleagues [25] identified a number of proteins involved in human nonsyndromic deafness. Nonsyndromic hearing loss is defined as loss occurring without other clinically detectable symptoms. It is characterized by mild-to-profound sensorineural hearing impairment, and it is caused by genetic mutations in proteins expressed in the middle ear, the inner ear or in both. So far, 93 protein-encoding genes linked to nonsyndromic hearing loss have been identified but not all of them are fully characterized [26]. Among the characterized nonsyndromic pathologies, one has been shown to result in excessive endocytosis and accumulation of caveolae. Prelingual nonsyndromic autosomic recessive deafness 1 (DFNB1) is caused by mutations in the Gap junction protein beta 2 (GJB2) gene encoding for the cochlear gap junction protein connexin 26 (CX26). One of the mutations induces degradation of the gap junction complexes through abnormal accumulation of cav1 and cav2 positive vesicles and increase of endocytosis leading to membrane retrieval [27].

In this study, it is shown that the administration of GTM to the SL pericytes induces changes in caveolae proteome profile. In particular, proteome changes occur in the association of Rab GTPase proteins, which are master controllers of the intracellular vesicular transport. Furthermore, we showed for the first time that SL pericytes express cav1 and cav2 but not cav3, independently of GTM exposure. Finally, we identified proteins known to be associated with nonsyndromic hearing loss in the caveolae of SL pericytes.

### Aims

The aim of this study is to investigate whether changes occur in the proteins profile associated with caveolae in GTM treated SL pericytes. A different protein profile in transport-specialized caveolae may indicate pathological changes potentially affecting the integrity of the BLB and ultimately contributing to hearing loss.

## Methods

### Cell isolation and culturing

SL pericytes were isolated from cochlea obtained from ImmortoMouse® (Charles River Laboratories, USA) carrying a conditional thermosensitive SV40 large T antigen functional at the permissive temperature of 33 °C but non-functional at the nonpermissive temperature of 39 °C [28, 29]. All experiments were conducted at the temperature of 39 °C.

Four-week-old mice were euthanized with CO_2_ and decapitated. Rapidly, the brain tissue was removed and both cochleae were extracted by fracturing the petrous portion of the temporal bone. Cochleae were then bathed in the ice cold transfer medium, containing Ca^++^ and Mg^++^ (HBSS Cellgro 21–023-CV, Mediatech, Inc. USA) and 20% Fetal Bovine Serum (FBS) (GIBCO 10091–130, Thermo Fisher Scientific, USA). The lateral wall tissue consisting of SL and SV was separated from the cochlear structure, and the two tissues further separated by using tweezers (Type 5 mini, super thin tips, DuMont, Electron Microscopy Science, USA) and a Zeiss Stereo Discovery V12 dissection microscope (Carl Zeiss Microscopy LLC, USA). Tissues were digested in a mixture of Dispase grade II protease (Roche Diagnostic, USA), collagenase type I and collagenase type IV (GIBCO, Thermo Fisher Scientific, USA) for 15 min at 37 °C in 5% CO_2_. Tissue digestion was stopped with 1 ml of neutralizing buffer consisting of DPBS without Ca^++^ and Mg^++^ supplemented with 10% FBS (GIBCO, Thermo Fisher Scientific, USA). The suspension was pipetted gently up and down in order to further separate the cells, then passed through a 70 μm cell strainer (Falcon™, Fisher Scientific, USA) and centrifuged (Beckman centrifuge GS 6R, USA) in ice cold neutralizing buffer for 10 min at 900 rpm. Cells were incubated in MV media without vascular endothelial growth factor (VEGF) to support pericyte growth (MV Media + kit, PromoCell, Heidelberg, Germany), in culture wells coated with gelatin (Cell Biologics Inc. Chicago, USA) and allowed to proliferate until 90% confluence was reached. CD31 and CD146 markers for endothelial cells and pericytes (anti-mouse CD31 antibody PE Cy7 Biolegend 1/100; and anti-mouse CD146 PE Biolegend 1/100), were used to sort the positive cells with a flow sorter FACSAria, (Harvard Medical School Flow Cytometry Core Facility, Boston, USA) (data not shown). Sorted cells were plated in vessels precoated with gelatin-based solution in MV media. Cells were confirmed as pericytes by flow cytometric analysis using the Accuri C6 Cytometer (BD Bioscience, USA). Cells tested negative for the endothelial cell marker anti-von Willebrand factor (vWF), sheep polyclonal Abcam, USA, with secondary antibody Alexa Fluor 488 donkey anti-sheep, Life technology, USA), and positive for the pericytes markers chondroitin sulfate proteoglycan 4 (NG2) (anti-NG2 antibody mouse monoclonal, Abcam, USA; secondary Alexa Fluor 488 goat anti-mouse, Life technology, USA) and Desmin (anti-desmin antibody rabbit monoclonal Abcam, USA; secondary Alexa Fluor 488 goat anti-rabbit, Life Technology, USA). Pericytes were further characterized as SL pericytes with the alpha-Smooth-Muscle-Actin (α-SMA), a protein absent in stria vascularis pericytes and a marker of SL pericytes (rabbit monoclonal anti-α-SMA, Abcam, USA; secondary was Alexa Fluor 488 goat anti-rabbit, Life Technologies, USA). SL pericyte cultures were expanded in gelatin coated T-75 flasks until a 80–90% confluence was reached, in a 5% CO_2_ environment at 39 °C and used according to the experimental design. When GTM (Gentamicin solution, AMRESCO, Solon, Ohio) was dissolved in MV media, sodium hydroxide (5 N solution, Fisher Scientific, USA) was added to the solution to adjust media pH.

### Caveolae isolation and protein analysis

Sample and gradient preparation: SL pericytes were detached from 150 cm^2^ flasks with Accutase cell detachment solution (BD Bioscience, USA) and washed twice in cold PBS. Caveolae/Raft isolation kit (Sigma–Aldrich, USA) was used according to the manufacturer’s instructions. All the procedures for the isolation of caveolae from SL pericytes: cell preparation, manipulation, as well as gradient preparation were conducted in a 4 °C cold room. The lysis buffer containing 1% Triton X-100 and protease inhibitor cocktail as well as the OptiPrep™ density gradient solutions used for the caveolae separation were prepared immediately before use and kept on ice. Tubes, pipets, pipet-tips, centrifuge and ultracentrifuge rotors used in the experiment were pre-chilled at 4 °C.

Briefly, cells were suspended in 100 μl of lysis buffer and kept on ice for at least 30 min. Aliquots of cell lysate were combined in a solution of 35% OptiPrep Density Gradient Medium and transferred to the bottom of a bell-top polyallomer ultracentrifuge OptiSeal tube (Beckman Coulter Inc. Brea, CA, USA). Four volumes of dilutions of OptiPrep™ in lysis buffer at the decreasing concentration of 30, 25 and 20% were then carefully layered from the bottom up of the OptiSeal tube containing the cell lysate, for a final volume of 4 ml. The Optiseal tubes were then centrifuged at 200,000 x g for 4 h at 4 °C with an Optima TLX ultracentrifuge equipped with a TLA 110 rotor (Beckman Coulter Inc. Brea, CA, USA). Once the ultracentrifugation was completed, the tubes were carefully extracted from the rotor and kept on ice. A pre-chilled manual pipette was used to collect eight or nine aliquots of approximately 0.5 ml each from the top down of each ultracentrifuge tube in a 4 °C environment. Aliquots were collected in ice cold microcentrifuge tubes and kept on ice.

### Dot-blot detection of caveolin-1 enriched aliquots

In order to identify the caveolae-rich aliquot, 3 μl from each gradient aliquot obtained from the ultracentrifugation was placed on a Polyvinylidene fluoride (PVDF) membrane (Immun-Blot® PVDF membrane, BIO-RAD Laboratories, USA) previously activated in 100% methanol (J.T. Baker, USA) and kept moist with filter paper rectangles (Mini Trans-Blot, BIO-RAD Laboratories Inc. USA) soaked in ultrapure MQ water. The aliquots’ drops were adsorbed on the membrane for a few minutes, and then incubated with 5% nonfat milk in PBS-T (Phosphate Buffered Saline with 1%Tween 20) for 1 h at room temperature and further incubated with anti-caveolin-1 antibody (Sigma-Aldrich, USA) overnight at 4 °C in 5% nonfat milk. The membrane was then washed with PBS-T and incubated for 1 h at room temperature with diluted secondary antibody (anti-rabbit IgG peroxidase conjugated, Life Technology, USA). Membrane chemiluminescence was developed with Clarity ECL substrate (Clarity Western ECL Substrate, BIORAD laboratories, USA) and visualized with Chemidoc MP Imaging System and software (BIORAD Laboratories, USA) [see Additional file 1]. The aliquots with the strongest signal for cav-1 were selected for protein separation and mass spectrometry analysis.

### Separation of proteins on SDS PAGE gel for mass spectrometry analysis

Caveolae were isolated and prepared for the mass spectrometry analysis in three independent experiments. A volume of 15 μl from the selected gradient aliquot was mixed with an equal volume of Laemmli buffer (BIO-RAD Laboratories, USA) and then loaded onto SDS precast gel TGX 4–15% (BIO-RAD Laboratories). Proteins were separated on the gel using a Mini-protean TGX system (BIO-RAD Laboratories, USA), bathed in running buffer solution (tris/glycine/SDS 1X buffer BIO-RAD Laboratories, USA). Gels were then incubated overnight in Coomassie blue for protein staining and fixation and washed in ultrapure water allowing several changes. The gel lanes were excised and separated into three fragments: roughly above 75 kDa, below 25 kDa and between 25 and 75 kDa. The fragments were then submitted to mass spectrometer. For the purpose of the analysis, the three bioinformatic files representing the fragments were subsequently reunited for the data analysis. When a protein was detected in more than one fragment, the peptide with the maximum counts was retained.

### Protein sequence analysis by LC-MS/MS

Excised gel fragments were cut into approximately 1 mm^3^ pieces and processed and analyzed by the Taplin Mass Spectrometry Facility (Harvard Medical School, Boston, MA)**.** Gel pieces were subjected to a modified in-gel trypsin digestion procedure [30]. Gel pieces were then washed and dehydrated with acetonitrile for 10 min followed by removal of acetonitrile. Pieces were then completely dried in a Speed-Vac. Rehydration of the gel pieces was done with 50 mM ammonium bicarbonate solution containing 12.5 ng/μl modified sequencing-grade trypsin (Promega, Madison, WI) at 4 °C. After 45 min, the excess trypsin solution was removed and replaced with 50 mM ammonium bicarbonate solution to just cover the gel pieces. Samples were then placed in a 37 °C room overnight. Peptides were later extracted by removing the ammonium bicarbonate solution, followed by one wash with a solution containing 50% acetonitrile and 1% formic acid. The extracts were then dried via vacuum centrifugation (~ 1 h). The samples were then stored at 4 °C until analysis. On the day of analysis the samples were reconstituted in 5–10 μl of HPLC solvent A (2.5% acetonitrile, 0.1% formic acid). A nanoscale reverse-phase HPLC capillary column was created by packing 2.6 μm C18 spherical silica beads into a fused silica capillary (100 μm inner diameter x ~ 25 cm length) with a flame-drawn tip [31]. After equilibrating the column each sample was loaded via a Famos autosampler (LC Packings, San Francisco CA) onto the column. A gradient was formed and peptides were eluted with increasing concentrations of solvent B (97.5% acetonitrile, 0.1% formic acid). As peptides eluted, they were subjected to electrospray ionization and then entered an LTQ Orbitrap Velos Pro ion-trap mass spectrometer (Thermo Fisher Scientific, San Jose, CA). The range of masses (m/z mass over charge) allowed in the search used was from 600 to 8000; charge z of the precursor ion 2, 3 and 4; fragment mass tolerance 1 Da; cleavage rule used: up to two missed cleavages. Modifications: differential: methionine oxidation; static: cysteine alkylation (iodoacetamide). Peptides were detected, isolated, and fragmented to produce a tandem mass spectrum of specific fragment ions for each peptide. Peptide sequences (and hence protein identity) were determined by matching protein databases with the acquired fragmentation pattern by the software program, Sequest-v28 (Thermo Fisher, San Jose, CA) [32]. All databases (Uniprot) include a reversed version of all the sequences and the data was filtered to between a one and 2 % peptide false discovery rate.

Only proteins detected in at least two of the three mass spectrometry runs were considered for further bioinformatic analysis. The latter was performed with R version 3.3.1 (R Core Team) and visualization done with VennDiagram R package [33] (https://www.r-project.org).

### Gene ontology enrichment analysis from identified proteins

The web-server interactive software tool ***G****ene* ***O****ntology en****RI****chment ana****L****ysis and visua****L****iz****A****tion tool* or GOrilla (http://cbl-gorilla.cs.technion.ac.il/) was selected for the gene enrichment analysis. The program enables GO enrichment analysis, identification and visualization of GO terms in unranked lists of genes for the three GO categories biological processes, cellular components, and molecular functions [34, 35]. The method identifies, independently for each GO term in the *Mus musculus* ontology, the threshold at which the most significant enrichment is obtained. Results are organized for a *p*-value threshold ranging from *p* < 10^− 3^ to *p* > 10^− 9^. The false discovery rate (FDR) q-value is associated with each term’s p-value and it is the corrected p-value for multiple testing, using the Benjamini and Hochberg method. The outputs are visualized in tables ranking the GO terms according to the p-value and corresponding FDR q-value, from the highest significant term down.

### Proteomaps functions analysis

The metabolic functions of the uniquely expressed proteins in the control and GTM data sets were visualized with the web-based interactive software Proteomaps www.proteomaps.net. The software visualizes the composition of proteomes with a focus on protein functions and abundance. Proteins are assigned to functions via modified KEGG (Kyoto Encyclopedia of Genes and Genomes) Orthology IDs, and are shown in Proteomaps as polygon-shaped tiles, with the area representing protein abundance. Proteomaps runs a modified algorithm for the construction of Voronoi treemaps to present polygons with variable sizes. The algorithm was implemented in the Paver software (DECODON, Greifswald, Germany) [36]. The mass spectrometry detection did not estimate the abundance of the protein obtained, therefore we assigned 1 as an arbitrary quantity for the analysis of the proteins loaded onto Proteomaps software, resulting in a map where areas represent the number of proteins in an assigned function.

### Nonsyndromic hearing loss protein segregating with caveolae

Protein encoding genes from the three mass spectrometer repeats were searched using the nonsyndromic Gene Homepage database [26] (http://hereditaryhearingloss.org). The site lists data and links for all known gene localizations and identifications for monogenic nonsyndromic hearing impairment.

### Western blot analysis

SL pericyte cells were incubated for 24 h with GTM at concentrations of 0, 1, 5 and 10 mg/ml. Cell lysates were prepared with 1X lysis buffer (RIPA buffer, Cell Signaling Technology). A protease and phosphatase inhibitor solution (Protease/Phosphatase inhibitor cocktail, Cell Signaling Technology, USA) was then added to the mixture. Proteins were separated in a 4–15% SDS-PAGE (Mini-protean TGX BIO-RAD Laboratories, USA) gel electrophoresis and transferred using a Trans-Blot Turbo Transfer system (BIO-RAD Laboratories, USA) using Trans Blot Turbo transfer pack 0.2 μm PVDF membranes (BIO-RAD Laboratories, USA). Membranes were blocked in 5% fat-free milk diluted in Tris-buffered saline with Tween (TBST; 0.1% Tween-20, 150 mM NaCl, 50 mM Tris, pH 7.5) for 1 h. The membranes were then incubated with purified monoclonal rabbit anti-caveolin 1 (Cell Signaling Technology, USA 1/1000), anti-caveolin 2 rabbit polyclonal and anti-caveolin 3 Rabbit polyclonal antibodies (Abcam, Boston, MA). Anti-Rab antibodies used were: anti-Rab3a (Cell Signaling Technology, USA, 1:1000), anti-Rab3b polyclonal (Novus Biologicals, USA, 1:500), Rab family antibody sampler kit (Cell Signaling Technology, USA, anti-Rab4; anti-Rab5; anti-Rab7; anti-Rab9a; anti-Rab11; 1:1000), anti-Rab6a polyclonal (GeneTex, USA, 1:100); anti-Rab6b polyclonal (Proteintech, Fisher Scientific, USA; 1:500), anti-Rab8a monoclonal (Abcam, USA, 1:500), anti-Rab13 polyclonal (Abcam, USA, 1:500), anti-Rab22a monoclonal (Abcam, USA, 1:1000), anti-Rab23 polyclonal (Abcam, USA, 1:500), anti-Rab3gap2, polyclonal (GeneTex, USA, 1:500). Monoclonal mouse anti-rabbit β-Actin HRP conjugated antibody (1:1000, Cell Signaling Technology, Danvers, MA, USA) was simultaneously incubated with the primary antibody. The membranes were maintained at 4 °C, with gentle shaking overnight then incubated with the secondary antibody (polyclonal goat anti-rabbit immunoglobulins/HRP 1:2000, Dako) for 1 h at room temperature. Bands were visualized using a Chemidoc MP Imaging system and software (BIO-RAD Laboratories, USA). The Rab proteins’ signal in each immunoblot was normalized to the corresponding signal for β-actin and the concentration of the Rab proteins was expressed as the relative quantity to the control.

### Annexin-V APC apoptotic assay

Cellular apoptosis was determined with an Annexin V assay (Annexin V Apoptosis Detection Kit, Affymetrix, eBioscience). SL pericytes were incubated for 24 h with GTM at concentrations of 0, 1, 5, and 10 mg/ml. The Annexin V assay was performed according to the manufacturer’s instructions. Cell fluorescence was measured with Accuri C6 (BD Biosciences, San Jose, CA) flow cytometer using FITC detector filters at 530 nm for annexin V and 600 nm for PI (Propidium Iodide). Data were analyzed using FlowJo software (FlowJo, LLC, Ashland, OR). Data resulted from four independent experiments for controls and three independent experiments for GTM-treated cells. Statistical computation was performed with a one-way ANOVA, followed by a Dunnett’s test (9 degrees of freedom) when data showed significance. Differences were considered significant for *p* values less than 0.05. Statistical tests were performed with R version 3.3.1 (R core team).

## Results

### Characterization of SL pericytes

**Fig. 2:**
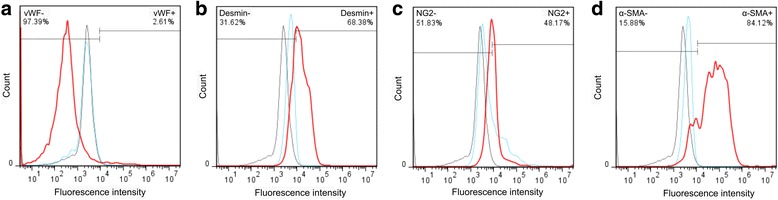
SL pericytes characterization. Flow cytometry analysis of cells obtained from the cochlear SL. The histograms show that 97.39% of the cells are negative for the expression of vWF, a validated marker for endothelial cells. Cells show positive expression for validated pericyte markers Desmin (68.4%), NG2 (48.2%), and α-SMA (84.1%). The detection of αSMA identifies pericytes from the SL, the only pericyte type in the microvasculature of the lateral wall to express the contractile protein. In the figure black histograms identify the unstained cells, blue histograms identify the isotype control and the red histograms identify the markers of interest

To exclude the presence of endothelial cells in the culture we used the endothelial cell marker vWF. VWF is a large glycoprotein expressed constitutively in endothelial cells and megakaryocytes. The flow cytometry analysis showed that nearly all cells (97.39%) did not express a signal for the vWF marker (Fig. 2a). Next, we used a panel of pericyte markers to precisely identify the cell type. The expression level of pericyte markers can be up- or down-regulated depending on various factors such as cell physiological status, pathological status and culture conditions [37]. The validated pericyte marker Desmin and NG2 were selected for the flow cytometry analysis and cell characterization. Data showed that 68.38% of the cells were positive for the antibodies against Desmin and 48.17% of the cell population was positive for the anti-NG2 antibody (Fig. 2b, c). We further proceeded to the identification of SL pericytes using the validated pericyte marker α-SMA. The stria vascularis pericytes, unlike other pericytes, do not express α-SMA [38] which is considered a marker for SL pericytes. Data from the flow cytometer analysis showed that 84.12% (Fig. 2d) of the cells were positive for α-SMA, identifying the population as pericytes of the spiral ligament microvasculature.

### Cav1 and cav2 expression in SL pericytes was not affected by gentamicin

**Fig. 3:**
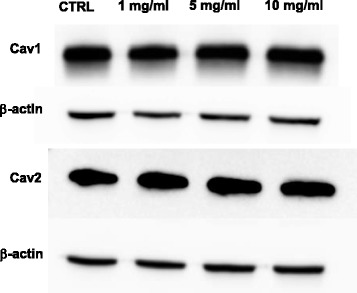
Caveolin-1 and caveolin 2 expression in SL pericytes is not affected by gentamicin. Western blot analysis of whole cell lysate from SL pericytes expressing caveoin-1 and cavolin-2. SL pericytes were incubated with increasing concentration of GTM for 24 h. The expression of cav-1 and cav-2 in the SL pericytes was not depleted by the GTM treatment

To understand if GTM challenge to the cells would deplete SL pericytes caveolins, cultures were incubated for 24 h with several concentrations of GTM and changes in the expression of caveolae proteins cav1 and cav2 were assayed with western blotting analysis. Cav1 is the constitutive protein of caveolae; its expression is essential and required for the formation of morphologically identifiable caveolae. Cav2 is usually coexpressed with cav1, most abundantly in endothelial cells, fibrocytes, and adipocytes, although its expression is regulated independently of cav1 and it is considered nonessential for caveolae formation, since lack of cav2 does not affect caveolae formation [39]. The western blot analysis showed that SL pericytes express abundant cav1 and cav2 (Fig. 3) but not cav3 (data not shown). The caveolins’ concentration was unaffected by the treatment from the lowest to the highest of the GTM concentrations used (Fig. 3).

### Gentamicin induced apoptosis in SL pericytes challenged for 24 h

**Fig. 4:**
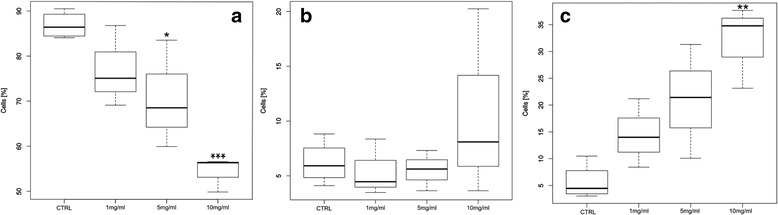
Gentamicin induced cell apoptosis is dose dependent. Box Plot graphs obtained from flow cytometry analysis of fluorescently labeled Annexin V and propidium Iodide (PI) SL pericytes. Cells were incubated for 24 h at increasing concentration of GTM (1, 5 and 10 mg/ml). **a** The percentage of live cells population showing a negative signal for either Annexin or PI staining, decreased significantly at the GTM concentration of 5 mg/ml (*p* < 0.049) and 10 mg/ml (*p* < 0.00079). **b** The Annexin positive PI negative population showed no significance after 24 h of GTM incubation at any of the GTM concentrations used. **c** Cell treated with 10 mg/ml GTM showed a marked significance (*p* < 0.0025) for late stage apoptosis. Data consisted of 4 independent experiments for controls and 3 independent experiment for GTM treated cell. Differences between controls and treated cells were analyzed with one-way ANOVA followed by a Dunnett’s test (9 degrees of freedom) when data showed significance. Differences were considered significant for *p* < 0.05. Statistical test were performed with R version 3.3.1 (R core team) *p < 0.05, **p < 0.01 ***p < 0.001

The apoptotic effect of GTM incubation on SL pericytes was analyzed by simultaneously double staining the cells with annexin-V and propidium iodide (PI) dye, distinguishing live cells, early stage apoptosis and late stage apoptosis. Annexin preferentially binds phosphatidylserine (PS), which in cell physiological condition is located in the inner leaflet of the plasma membrane. In early stage apoptotic cells, PS is translocated to the extracellular membrane leaflet where it is detected by fluorescently labeled Annexin V. Positive staining of chromatin by PI occurs in the late stage apoptosis when the cell membrane loses integrity allowing PI in the interior of the cell. Data from the flow cytometer analysis are shown in Fig. 4. The percentage of live cells population showing a negative signal for either Annexin or PI staining, decreased significantly at the GTM concentration of 5 mg/ml (*p* = 0.049) and 10 mg/ml (*p* = 0.00079). The Annexin positive PI negative population showed no significance after 24 h of GTM incubation at any of the GTM concentrations used. Cells treated with 10 mg/ml GTM showed a significant increase (*p* = 0.0025) at late stage apoptosis. The results showed that 24 h incubation with GTM at the concentration of 10 mg/ml stressed the cells, significantly inducing apoptosis. SL pericytes incubated at lower GTM concentrations showed signs of stress, such as a decrease in cell number, without a significant increase of cell apoptosis. Based on these results, we selected the concentration 5 mg/ml of GTM to stress the cells without inducing a significant level of apoptosis, in order to identify the specific proteome in challenged cells.

### Analysis of the caveolae proteome in SL pericytes

Proteins segregating with caveolae extracted from control and GTM-treated SL pericytes were analyzed by mass spectrometry. Venn diagrams were used to visualize the similarities and differences in the caveolae proteome in GTM treated and untreated SL pericytes in the three mass spectrometry experiments. Overall 3230 proteins were identified considering all the control runs and 3902 proteins considering all the GTM runs. 23.4% of proteins were found common to all control runs and 22.5% of proteins were found common to all GTM runs. In order to obtain a stringent result and confidently identify the uniquely expressed protein in control and GTM treated datasets, proteins found in at least two of the three mass spectrometry runs were considered for the bioinformatic analysis. Proteins that only showed in one out of the three mass spectrometry repeats either in control or in the GTM dataset were removed, leaving 1682 proteins in the control runs and 2379 in the GTM runs. Of these, 251 proteins (15%) uniquely segregated with caveolae in control, 948 proteins (40%) uniquely segregated with caveolae in GTM, and 1431 proteins were common to both datasets [see Additional file 2].

### Enrichment analysis of proteins segregating with caveolae in gentamicin challenged cells

Protein encoding genes from the control and GTM-treated datasets that appeared in two out of three mass spectrometry repeats were considered for the enrichment analysis. The GTM dataset was the target group and the control dataset plus the GTM dataset, were chosen as background group. Gene ontology terms with q-value below 0.05 are listed in Tables 1A and B. The enrichment analysis was performed for the GO categories “Molecular function” which identify the molecular activity of genes products, “Cellular component” which indicates where gene products are active and “Biological process” which identifies pathways and larger processes comprising the activities of multiple gene products.

**Table 1:** A and B. Enriched terms for the ontologies “Biological process” and “Cellular component” in gentamicin treated cells. Proteins that appeared in two out of three mass spectrometry repeats were considered for the analysis. Proteins segregating with caveolae in the GTM treated cells dataset where selected as the target for the GOrilla enrichment analysis. The control dataset plus the GTM dataset were chosen as background group in the analysis. All terms with FDR q-value below 0.05 are listed in Tables 1A and B. (A) The GO category “Cellular Process” showed significant enrichment for processes associated with transport and localization, and associated to metabolic, bioenergetic and biosynthetic processes. (B) The GO category “Cellular component” showed significant enrichment for terms participating in three main groups, one comprising terms referring to cell cytoplasm and organelles, the other comprising terms referring to extracellular components and vesicles, and a third group comprising terms referring to cell membrane and membrane/protein interactions.

A GO biological process			
GO term	Description	FDR q-value	Enrichment (N, B, n, b)
GO:0044710	single-organism metabolic process	3.50E-06	1.07 (2597,532,2348,515)
GO:0044281	small molecule metabolic process	1.65E-05	1.09 (2597,323,2348,317)
GO:0045184	establishment of protein localization	9.64E-03	1.06 (2597,376,2348,362)
GO:0006810	transport	8.07E-03	1.05 (2597,709,2348,670)
GO:0015031	protein transport	1.23E-02	1.07 (2597,351,2348,338)
GO:0008104	protein localization	1.62E-02	1.06 (2597,431,2348,412)
GO:0055114	oxidation-reduction process	1.71E-02	1.08 (2597,214,2348,209)
GO:0051179	localization	1.85E-02	1.04 (2597,793,2348,745)
GO:0042592	homeostatic process	1.71E-02	1.08 (2597,193,2348,189)
GO:0043436	oxoacid metabolic process	1.85E-02	1.09 (2597,172,2348,169)
GO:0019752	carboxylic acid metabolic process	1.68E-02	1.09 (2597,172,2348,169)
GO:0006082	organic acid metabolic process	2.16E-02	1.08 (2597,187,2348,183)
GO:0051234	establishment of localization	2.27E-02	1.04 (2597,733,2348,689)
GO:0044711	single-organism biosynthetic process	2.62E-02	1.08 (2597,183,2348,179)
B GO Cellular component			
GO term	Description	FDR q-value	Enrichment (N, B, n, b)
GO:0044444	cytoplasmic part	1.91E-08	1.03 (2597,1603,2348,1499)
GO:0031982	vesicle	6.31E-07	1.05 (2597,1045,2348,988)
GO:0043230	extracellular organelle	7.51E-07	1.05 (2597,942,2348,893)
GO:1903561	extracellular vesicle	5.63E-07	1.05 (2597,942,2348,893)
GO:0070062	extracellular exosome	4.86E-07	1.05 (2597,941,2348,892)
GO:0031988	membrane-bounded vesicle	4.37E-07	1.05 (2597,1000,2348,946)
GO:0044421	extracellular region part	3.38E-06	1.04 (2597,982,2348,927)
GO:0098796	membrane protein complex	1.24E-05	1.09 (2597,272,2348,267)
GO:0044425	membrane part	1.25E-04	1.05 (2597,781,2348,738)
GO:0016020	membrane	4.63E-04	1.03 (2597,1470,2348,1363)
GO:0044432	endoplasmic reticulum part	2.25E-03	1.09 (2597,153,2348,151)
GO:0005829	cytosol	1.14E-02	1.05 (2597,397,2348,378)
GO:0031090	organelle membrane	2.65E-02	1.05 (2597,435,2348,412)
GO:0005783	endoplasmic reticulum	2.77E-02	1.06 (2597,312,2348,298)
GO:0005739	mitochondrion	2.82E-02	1.04 (2597,508,2348,479)
GO:0098588	bounding membrane of organelle	4.45E-02	1.06(2597,231,2348,222)

The category “Biological Process” (Table 1A) showed a strongly significant enrichment for terms participating into two groups. One group comprising the processes associated with transport and localization, and the other comprising terms referring to metabolic, bioenergetic and biosynthetic processes. In the first group the highest significance was reached by the terms “Transport” (GO:0006810; FDR q-value 8.07X10^− 3^) and “Establishment of protein localization” (GO: 0045184; FDR q-value 9.64X10^− 3^), followed by the terms “Protein transport” (GO: 0015031), “Protein localization” (GO:0008104), “Localization” (GO: 0051179) and “Establishment of localization” (GO: 0051234) all with FDR q-value< 0.05.

In the second group, the highest significance was reached by the terms “Single organism metabolic process” (GO:0044710; FDR q-value 3.50X10^− 6^) and “Small molecule metabolic process” (GO:0044281; FDR q-value 1.65X10^− 5^) followed by the terms “Oxidation reduction process” (GO:0055114), “Homeostatic process” (GO:0042592), “Oxoacid metabolic process” (GO:0043436), “Carboxylic acid metabolic process” (GO:0019754), “Organic acid metabolic process” (GO:0006082) and “Single organism biosynthetic process” (GO:0044711), all with FDR q-value< 0.03.

Terms for GO “Cellular component” (Table 1B) showed a strongly significant enrichment for terms participating in three main groups: one group comprising the cell cytoplasm and organelles, another comprising terms referring to extracellular components and vesicles, and finally, a group comprising terms referring to cell membrane and membrane/protein interactions. In the first group the highest significance was reached by the term “Cytoplasmic part” (GO: 0044444 FDR q-value 1.91X10^− 8^), followed by the term “Endoplasmic reticulum part” (GO:0044432; FDR q-value 2.25X10^− 3^). The terms “Cytosol” (GO:0005829),“Organelle membrane” (GO:0031090), “Endoplasmic reticulum” (GO:0005783) and “Mitochondrion” (GO:0005739) followed, all with a FDR q-value< 0.03.

In the second group, the highest significance was reached by the terms “Extracellular organelle” (GO:0043230), “Vesicle” (GO:0031982), “Extracellular vesicle” (GO: 1,903,561), “Extracellular exosome” (GO:0070062), “Membrane bounded vesicle” (GO:0031988) all with FDR q-value 10^− 7^ followed by the term “Extracellular region part” (GO:004442 FDR q-value 3.38X10^− 6^).

In the third group, the highest significance was reached by the term “Membrane protein complex” (GO:0098796 FDR q-value 1.24X10^− 5^) followed by the terms “Membrane part” (GO:0044425) and “Membranes” (GO:0016020) FDR q-value 10^− 4^, followed by the term “Bounding membrane of organelle” (GO:0098588 FDR q-value 4.45X10^− 2^).

Interestingly, none of the terms in the category “Molecular function” reached a statistically significant enrichment (FDR q-value > 0.05).

The analysis of the enriched terms in the GTM dataset confirms the activity of caveolae as a microdomain mostly involved in protein transport and participating in movement, localization and tethering of proteins to membranes and vesicles in the cell cytosolic compartments. In pathological conditions, such as when cells are challenged with GTM, caveolae significantly participate in pathways involving interactions with extracellular vesicles, cytoplasmic vesicles and organelles, in particular directed toward endoplasmic reticulum and mitochondria. The enrichment of metabolic bioenergetic and biosynthetic processes, together with the enrichment of endoplasmic reticulum and mitochondria cellular component, reveals the importance of caveolae participation in the activation and maintenance of biosynthesis and metabolic pathways when the cell undergoes to pathological stress. These findings establish a new and important role for caveolae as essential microdomains for the metabolism and biosynthetic processes in SL pericytes.

### Enrichment analysis of proteins uniquely segregating with caveolae in gentamicin challenged cells

In order to discover which categories were enriched in the proteins specifically expressed during the GTM challenge and suppressed in untreated cells, the 948 proteins uniquely segregating with caveolae in the GTM dataset were chosen as the target for the GOrilla enrichment analysis. Together, the control and GTM datasets were used as background. The GO terms were ranked in a table according to the FDR q-value with value below 0.05 considered significant.

The enrichment of the protein encoding gene dataset uniquely expressed in GTM challenged cells, reached significance only in the GO category “Molecular function” (Table 2). The GO terms, “Transferase activity transferring phosphorus containing groups” (GO:0016772; FDR q-value 6.34X10^− 3^), “Transferase activity” (GO: 0016740; FDR q-value 1.70X10^− 2^), and “Phosphotransferase activity alcohol group as acceptor” (GO:0016773; FDR q-value 4.09X10^− 2^) were significantly enriched in this category.

**Table 2:** Enriched terms in proteins uniquely segregating with caveolae in gentamicin challenged cells. The 948 proteins uniquely segregating with caveolae in GTM treated cells where selected as the target group for the GOrilla enrichment analysis. The control dataset plus the GTM dataset were chosen as background group. GO terms with a FDR q-value *p* < 0.05 were considered in the table. “Molecular function” was the only in the GO category reaching significance. The majority of the proteins associated with the enriched terms are involved in protein kinases activity

Molecular function			
GO term	Description	FDR q-value	Enrichment (N, B, n, b)
GO:0016772	transferase activity, transferring phosphorous-containing groups	6.34E-3	1.57 (2597.121, 932, 68)
GO:0016740	transferase activity	1.70E-2	1.32 (2597, 289, 932,137)
GO:0016773	phosphotransferase activity, alcohol group as acceptor	4.09E-2	1.60 (2597, 82, 932, 47)

The majorities of the proteins associated with the enriched terms are involved in protein kinases activity such as, cyclic adenosine monophosphate (cAMP) dependent, protein serine/threonine kinase activity, protein kinase C (PKC), calmodulin (CaM) and mitogen-activated protein (MAP) kinases and proteins involved in cysteine endopeptidase activity. The protein kinases listed regulate several aspects of cell functionality. Represented functions span from transport activities such as endocytosis and Rab proteins trafficking to regulation of cell cycle control, progression, arrest and volume control. Represented functions regulate cell proliferation, motility and morphology, cytoskeletal reorganization, expression of stress fibers, cell adhesion and nuclear signaling. Represented are also kinases active in inflammation and apoptosis processes such as MAPK, c-Jun N-terminal kinase (JNK) signaling pathways, transcription factors such as NFkB (nuclear factor kappa-light-chain-enhancer of activated B), proinflammatory cytokines, protein involved in the inflammasome assembly and in apoptotic pathways. The list includes also kinases participating in the calcium metabolism and proteins participating in the cell energy metabolism, in metabolism control and in glycolysis, such as hexokinase glycolytic enzymes, energy reduction sensing enzymes adenosine monophosphate-activated protein kinase (AMPK) and proteins participating in the regulation of lipid metabolism and lipid synthesis, catabolism control, autophagy and cell growth.

These data show cells boost pathways for cell survival and apoptosis that and may enhance metabolism and biosynthesis in an attempt to maintain the cell functioning and survival during GTM exposure. Furthermore these data show caveolae acting together with cell kinases to activate pathways that are essential for cells vital processes during GTM exposure.

### Enrichment analysis of proteins uniquely segregating with caveolae in untreated cells

In order to discover which GO categories were suppressed when cell were exposed to GTM, the 251 protein encoding genes uniquely segregating with caveolae in the control dataset were chosen as the target. For this analysis, together, control and GTM datasets were used as background. All terms with FDR q-value below 0.05 were considered significant. Data are presented in Tables 3A, B, C where GO terms reaching a significant enrichment up to FDR q-value of 10^− 5^ are listed. The full list of significantly enriched terms is presented in the additional files [See Additional file 3].

**Table 3:** A, B and C. Enrichment analysis of proteins uniquely segregating with caveolae in untreated cells. The 251 proteins uniquely segregating with caveolae in untreated cells where selected as the target group for the GOrilla enrichment analysis. The control dataset plus the GTM dataset were chosen as background group. GO terms with a FDR q-value p < or = 10^− 5^ were considered in the table. The complete list of significantly enriched GO terms to *p* < 0.05 is presented as additional file [See Additional file 3]. The enrichment showed significance for terms in the categories “Biological process”, “Cellular component” and “Molecular function”. The enriched terms showed the suppressed activities and functions in the cells once GTM is administered.

A Biological process			
GO term	Description	FDR q-value	Enrichment (N, B, n, b)
GO:0090304	nucleic acid metabolic process	2.59E-10	1.96 (2597,537,249,101)
GO:0016070	RNA metabolic process	3.1E-10	2.06 (2597,455,249,90)
GO:0006139	nucleobase-containing compound metabolic process	1.86E-5	1.61 (2597,673,249,104)
GO:0046483	heterocycle metabolic process	4.34E-5	1.57 (2597,704,249,106)
GO:0006725	cellular aromatic compound metabolic process	6.41E-5	1.56 (2597,694,249,104)
GO:0006351	transcription, DNA-templated	7.04E-5	2.23 (2597,210,249,45)
GO:0097659	nucleic acid-templated transcription	6.03E-5	2.23 (2597,210,249,45)
GO:0010468	regulation of gene expression	7.14E-5	1.66 (2597,539,249,86)
GO:0032774	RNA biosynthetic process	8.61E-5	2.19 (2597,214,249,45)
GO:0006396	RNA processing	9.71E-5	2.13 (2597,230,249,47)
B Cellular component			
GO term	Description	FDR q-value	Enrichment (N, B, n, b)
GO:0044428	nuclear part	1.15E-6	1.54 (2597,855,249,126)
GO:0005634	nucleus	4.66E-6	1.38 (2597,1160,249,154)
C Molecular function			
GO term	Description	FDR q-value	Enrichment (N, B, n, b)
GO:0003676	nucleic acid binding	5.68E-10	1.70 (2597,779,249,127)
GO:0003677	DNA binding	1.58E-7	2.36 (2597,243,249,55)
GO:0000975	regulatory region DNA binding	8.02E-7	3.56 (2597,79,249,27)
GO:0044212	transcription regulatory region DNA binding	6.01E-7	3.56 (2597,79,249,27)
GO:0001067	regulatory region nucleic acid binding	9.13E-7	3.48 (2597,81,249,27)
GO:0043565	sequence-specific DNA binding	1.34E-5	3.09 (2597,91,249,27)
GO:1990837	sequence-specific double-stranded DNA binding	4.73E-5	3.48 (2597,63,249,21)

The enrichment showed significance for terms in the categories “Biological process”, “Cellular component” and “Molecular function” (Tables 3A, B, C and Additional file 3). The category “Biological process” (Table 3A) showed a strongly significant enrichment for terms participating into two main groups. The first one comprising metabolic processes with the higher significance for the terms “Nucleic acid metabolic process” (GO:0090304; FDR q-value 2.59X10^− 10^) and “RNA metabolic process” (GO:0016070; FDR q-value 3.1X10^− 10^), followed by the terms “Nucleobase-containing compound metabolic process” (GO:0006139), “Heterocycle process” (GO:0046483) “Cellular aromatic compound metabolic process” (GO:0006725), and “RNA biosynthetic process”, all of them with a FDR q-value 10^− 5^. The second group comprises proteins involved in transcription and genetic information processing such as the terms “Regulation of gene expression” (GO:0010468), “Transcription DNA template” (GO:0006351), and “Nucleic acid template transcription” (GO:0097659) and “RNA processing” all of them with a FDR q-value 10^− 5^.

The ontology “Cellular component” (Table 3B) had the higher enrichment for the terms.

“Nuclear part” (GO:0044428; FDR q-value 1.15X10^− 6^) and “Nucleus” (GO:0005634; FDR q-value 4.66X10^− 6^). Proteins grouped under the terms accounted mainly for proteins active in the spliceosomal complex.

The ontology “Molecular function” had significantly enriched terms involved in RNA and DNA binding. The higher significance was reached by the terms “Nucleic acid binding” (GO:0003676; FDR q-value 5.68X10^− 10^) followed by the terms “DNA binding” (GO:0003677; FDR q-value 1.58X10^− 7^), “Regulatory region nucleic acid binding” (GO:0000975), “Transcriptional regulatory region DNA binding” (GO:0044212), and “Regulatory region nucleic acid binding” (GO:0001067), with a FDR q-value 10^− 7^. The term “Sequence-specific DNA binding” (GO:0043565) and “Sequence-specific double stranded DNA binding” with FDR q-value 10^− 5^. The major difference showed by the enrichment analysis in the untreated cells versus the GTM exposed cells is that a bulk of activities involving genetic information processing, occurring between the nucleus and cytoplasmic parts were suppressed. The genetic information processing is mainly represented by the spliceosome activity, DNA/ RNA metabolic processes positive/negative regulation of DNA templates, RNA splicing, and ribonucleoprotein activities. These pathways are significantly suppressed once the cells were exposed to GTM. Quite interestingly, these findings may show the important role of caveolae in transferring genetic information from the nucleus to the cytoplasm participating in the cell genetic information processing. The data show also the versatility of usages of caveolae by the cells in physiological or pathological conditions, switching the main activities from nucleus associated and cytoplasm transport, to participating in energy metabolism and activation of pathways under stress conditions.

### Proteomaps of proteins uniquely segregating with caveolae in control and gentamicin exposure cells

**Fig. 5:**
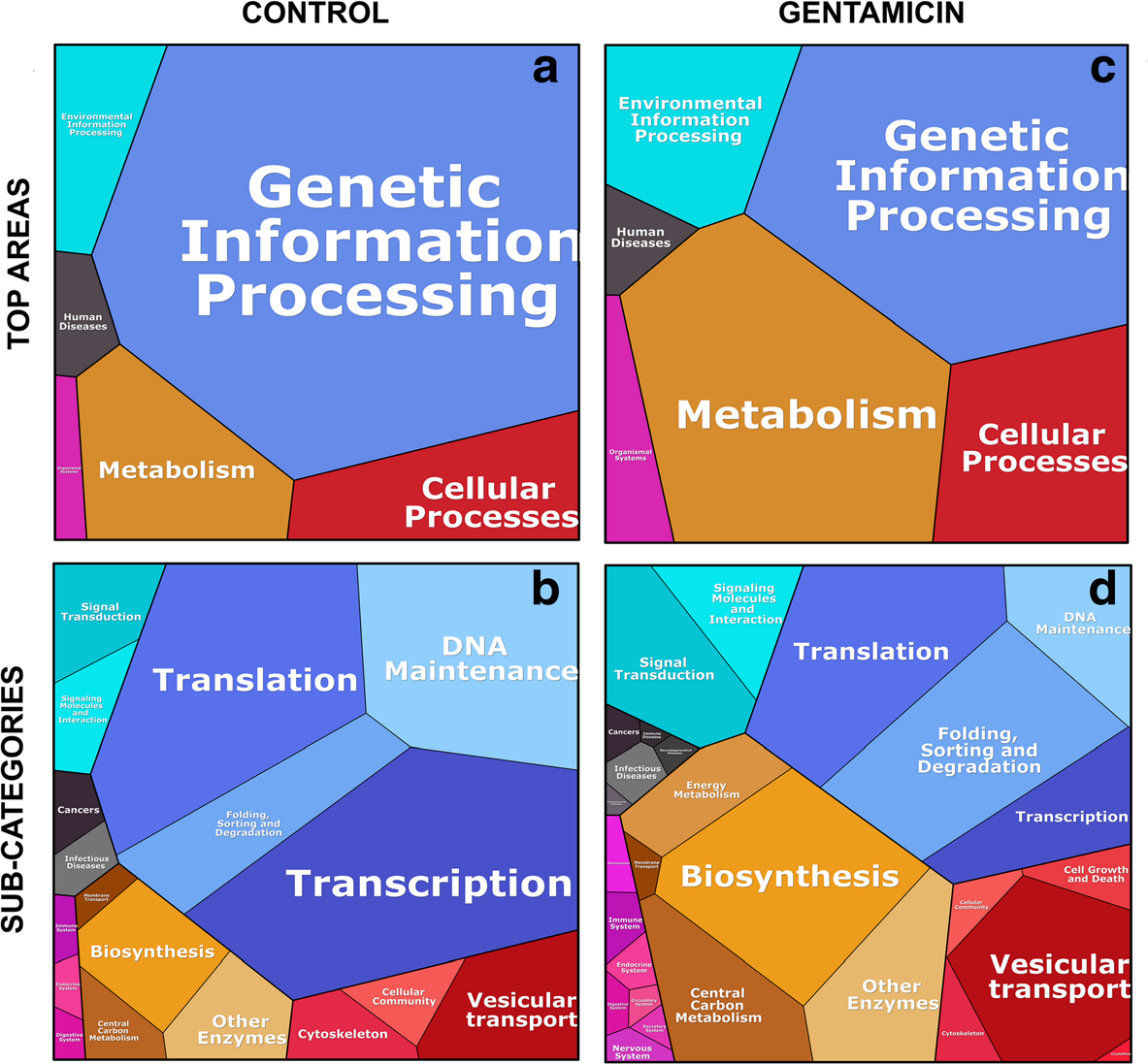
Comparative visualization of the proteins uniquely segregating with caveolae in control and gentamicin treated cells. Panels **a** and **c** show the top level area polygons for control and GTM datasets respectively. Panels **b** and **d**, show the division of the top area polygons in sub-categories for the two datasets. The visualization shows a dramatic difference in the polygon sizes in GTM treated vs control cells. The “Genetic information processing” polygon shows a reduction of “Translation”, “Transcription” and “DNA maintenance” processes and an increase in the “Sorting folding and degradation processes”. All the sub-categories in the “Metabolism” polygon double the size in GTM treated cells as compared to the untreated ones and a new subcategory “Energy metabolism” appeared. The doubling in size of the “Cellular processes” in GTM treated cells, mostly accounts for by the increase of the “Vesicular transport” process and the introduction of a new subcategory “Cell growth and death”. The subcategory “Signal transduction”, accounts for the increase of the top area Environmental information processes. Control and gentamicin top area polygons panel **A** and **C** in the figure, from the top left corner clock wise are: Environmental information processes, Genetic information processes, Cellular processes, Metabolism, Organismal system, Human diseases. Control: Subcategories panel **B** from the top left corner clock wise are: Signaling molecule and interaction, Signal transduction, translation, DNA maintenance, Folding sorting and degradation, Transcription; Vesicular transport cellular community cytoskeleton; Other enzymes, Central carbon metabolism, Biosynthesis, Membrane transport; Digestive system, Immune system, Endocrine system; Cancer, Infectious diseases. Gentamicin: subcategories panel **D**, from the top left corner clock wise are: Signal transduction, Signaling molecule and interaction; Translation, DNA maintenance, Folding sorting and degradation, Transcription; Cell growth and death, Vesicular transport Cytoskeleton, Cellular community; Other enzymes, Central carbon metabolism, Biosynthesis, Membrane transport Energy metabolism; Nervous, Excretory, Circulatory, Digestive, Immune, Endocrine systems, Development; Cardiovascular, Infectious diseases Cancer, Immune and Neurodegenerative diseases

In order to visualize the differences between the proteins uniquely expressed in control and GTM exposed cells and in order to confirm the results obtained in the enrichment analysis with an additional bioinformatic tool, we uploaded the 948 and 251 proteins from the control and GTM datasets respectively to the web-available interactive software Proteomaps (Fig. 5 and Additional file 4). First, the gene names from the mass spectrometry runs were looked up in UniProt / KEGG Orthology (mouse, 26,008 entries) to get gene IDs acceptable to the mouse branch of Proteomaps. Areas in the Proteomaps are proportional to the number of proteins in the category as represented in the modified KEGG orthology. To create a Proteomap, a total area is first divided into color-coded polygons representing the top-level categories.The top-level category areas are then subdivided into subcategories where functionally related proteins share common regions. All proteins entered in the Proteomaps analysis were given an arbitrary quantity value = 1. In the control dataset 39 out of 251 proteins were not recognized by the KEGG orthology. From the 212 submitted proteins, 178 were supported by Proteomaps treemap, corresponding to 77% coverage. In the GTM dataset 99 out of 948 proteins were not recognized by the KEGG orthology. From the 849 submitted proteins 599 were supported by Proteomaps treemap, corresponding to 70.6% coverage.

In the control top-level areas of the Proteomaps the category “Genetic information processing” had the largest polygon (Fig. 5a). In the control the polygons representing “Metabolism”, “Cellular processes” and “Environmental processes” were smaller and similar in size. In GTM exposed cells (Fig. 5c) the Proteomaps visualization showed a dramatic difference in the polygon sizes as compared to the control sizes. In the top-level areas the polygons “Metabolism”, “Cellular processes” and “Environmental information processes” double in size as compared to the control polygons, while the polygon “Genetic information processes” in the GTM top-level area (Fig. 5c) dramatically decreases as compared to the same category in the control dataset.

The subdivision of the top-level areas into subcategories in Figs. 5b and 4d, shows more detailed information of the processes affected and enhanced in control and GTM exposed cells. GTM exposure dramatically suppress the proteins participating in the subcategories “Translation”, “Transcription” and “DNA maintenance”, while “Folding sorting and degradation” processes (within the “Genetic information processes” top polygon) double in size as compared to the control. Furthermore, the sub-categories “Biosynthesis” and “Central carbon metabolism” double their size in GTM exposed cells as compared to control and a new sub-category, “Energy metabolism”, was detected. GTM affects cell signaling; in the top area “Environmental information processing”, the subcategory “Signal transduction” and “Signaling molecule and interaction” double in size as compared to control. Finally, GTM exposed cells enhanced vesicular transport. In top area “Cellular processes” the subcategory “Vesicular transport” doubles in size as compared to control and the subcategory “Cell growth and death”, not represented in control, appeared.

These results confirm the previously obtained results using the GOrilla enrichment analysis. Overall, the GTM exposure resulted in a dramatic decrease in the genetic information processing and a dramatic increase in cell metabolism both aerobic and anaerobic, and an increase in biosynthesis, signal transduction and vesicular transport. These outcomes, together with the representation the subcategory “Cell growth and death”, suggest that cells are responding to stress and activating survival mechanisms, which include increase in biosynthesis, energy metabolism, and proteins degradation on one side, and apoptosis on the other. GTM dramatically reduced the number of proteins involved in transcription and DNA maintenance activities. The increase in vesicular transport showed that caveolae is a major player in all of those processes. Finally, the similarity of the results obtained in the two distinct bioinformatic analyses, Proteomaps and GOrilla, confirm the robustness of these results.

### Transporter Rab GTPases segregating with caveolae in the SL pericytes

**Fig. 6:**
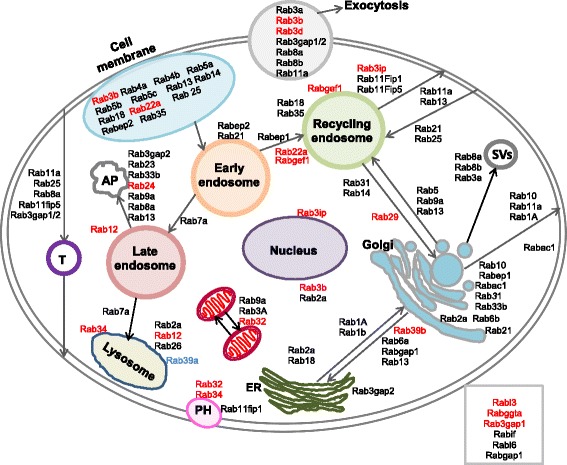
Rab proteins segregating with caveolae in control and gentamicin challenged cells. All Rab proteins appearing in the 3 MS runs are shown in the map. Black characters are Rabs segregating with caveolae in both GTM treated and untreated cells. Rab proteins found only in the GTM dataset are represented with red characters. The Rab protein segregating with caveolae only in the untreated cells is represented with blue character. Functions and localization of Rabs in the box on the lower right are unknown or inferred. Rab proteins localization and function in the map are based on the UniProt description. Secretory vesicles (SVs); transcytosis (T) autophagosome (AF); phagocytosis (PH)

The bioinformatic analysis showed that the GTM exposure boosted the vesicular transport in SL pericytes. We then focused our attention on Rab GTPases a group of transporter proteins often found associated to the caveolae and known to regulate trafficking in all cellular compartments. Table 4 shows all the Rab proteins segregating with caveolae in each of the three mass spectrometry repeats for control and GTM exposed cells. In the whole caveolae proteome obtained from the three mass spectrometry repeats, 36 Rab proteins were found associated with caveolae in the control dataset and 49 Rab proteins were associated with caveolae in the GTM dataset. Fourteen Rab proteins, shown in red under the GTM columns: Rab3b, Rab3d, Rab3gap1, Rab3ip, Rab12, Rab22a, Rab24, Rab29, Rab32, Rab 34, Rab 39b, Rabgef1, Rabggta, and Rabl3 were found uniquely expressed in the GTM exposed cells. Only one protein, Rab39a, was uniquely expressed in the control cells. An enrichment analysis was performed in order to understand if the GTM exposed cells were significantly enriched for the Rab proteins. Rabs appearing in two out of three mass spectrometry repeats were selected. The enrichment was determined using the hypergeometric function in R, phyper, for the pool of proteins in control and exposed cells. The pool of Rab proteins expressed in the caveolar fraction during the GTM challenge was significant with *p* = 0.032. While the pool of Rab proteins expressed in the control proteome of caveolae was not significantly enriched (*p* = 0.78). Figure 6 shows the localization and the interactions of the transporter Rab proteins with the cytoplasmic and membrane components in the cell. The figure is based on the Uniprot descriptions of the corresponding Rab proteins.

**Table 4:**
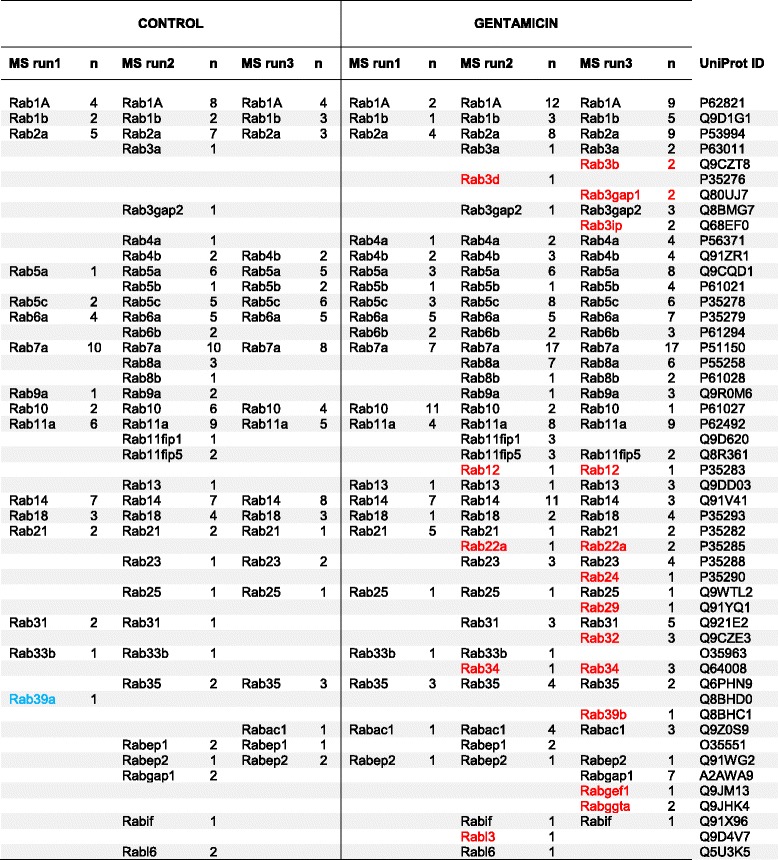
Rab proteins segregating with caveolae in the mass spectrometry analysis in gentamicin treated and untreated cells

Rab proteins segregating with caveolae in the three mass spectrometry (MS) experiments are listed in the table. The UniProt identifiers are given in the table for each Rab protein as well as the highest number of unique peptides (n) identifying the proteins. Black characters are Rabs segregating with caveoale in both GTM treated and untreated cells. Rab proteins found only in the GTM dataset are represented with red characters and the Rab protein found only in the control dataset is represented with blue.

Overall, the data shows that Rab proteins segregating with caveolae are involved in trafficking from the cell membrane to the endosomes network, from the endosomes to the lysosome and the autophagosome, and between the endoplasmic reticulum and the Golgi vesicles. Rabs represented are also involved in exocytosis, transcytosis and in secretory vesicles, transport to mitochondria and phagosome. Interestingly, Rabs uniquely found in GTM challenged cells participate in exocytosis pathways (Rab3b and Rab3d), in the phagocytic pathway (Rab32 and Rab34) and transport to mitochondria (Rab32 and Rab3A), in pathways involving trafficking from cell membrane to endosomes (Rab3b and Rab22a), in Golgi trafficking to cell membrane and endocytic pathways (Rab39b and Rab29), in trafficking from late endosome to lysosome and autophagosome pathways (Rab24, Rab12, Rab34) and finally in transport in the perinuclear area (Rab3ip and Rab3b). The functions and localizations of a few Rabs segregating with caveolae expressed in both datasets (Rabif and Rabl6) and segregating only in GTM dataset (Rabl3, Rabggta, Rab3gap1) are unknown.

GTM exposure significantly enhanced the Rabs associated with caveolae in GTM exposed vs control cells. The segregation of specific Rab proteins with caveolae in GTM exposed cells suggests a change in trafficking toward specific pathways. Rab proteins associated with caveolae in GTM treated cells have functional activity with the autophagosome and the lysosome pathways, showing a preference in the activity of Rab transport toward protein degradation and endosomal trafficking. GTM exposure enhanced the Rab proteins trafficking from the cell membrane to endosomal network, to the recycling endosome and toward exocytosis. Pathways of Rab proteins trafficking toward and from the Golgi apparatus and in the mitochondrial network and perinuclear area were also represented. The increased association of Rab proteins with caveolae in GTM exposed cells and the significant enrichment of the pool of Rabs expressed in the GTM exposed may be used as a marker for cellular stress.

### Rab proteins immunoblotting quantification

A pool of Rab proteins was selected for immunoblot quantification from the list of the Rabs isolated with caveolae and identified with LC MS/MS. Rabs selected for the analysis were chosen based on larger presence of a specific Rab protein in the GTM group versus the control group in the six LC MS/MS runs, as shown in Table 4. The Rab proteins selected were Rab3a, Rab3b, Rab3gap2, Rab4, Rab5, Rab6a, Rab6b, Rab7, Rab8a, Rab9, Rab11, Rab13, Rab22a, Rab23.

**Fig. 7:**
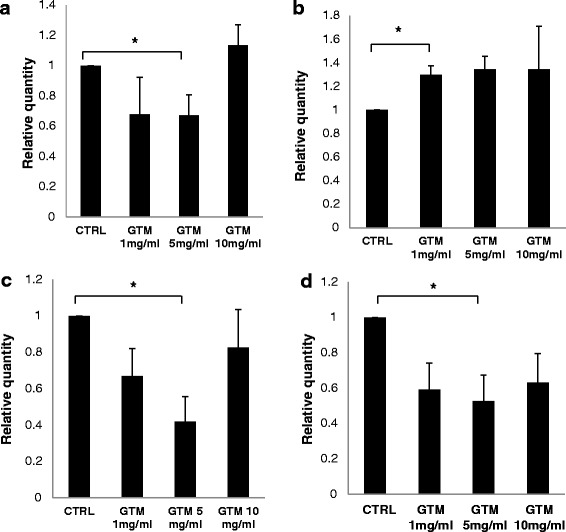
Concentration effect of GTM on Rab proteins in SL pericytes. SL pericytes were incubated with increasing concentrations of GTM (1 mg/ml, 5 mg/ml,10 mg/ml GTM) for 24 h. Immunoblots were obtained for each Rab protein from the whole cell lysate. Protein quantification is expressed as the relative quantity to the control for each Rab. Each graph is the result of *n* = 6 independent experiments for Rab8a (**a**) and Rab13 (**c**) and *n* = 4 independent experiments for Rab9 (**b**) and Rab 3gap2 (**d**). SEM was calculated for each group. Two tailed, paired Student’s ttest was used for statistical analysis with significance set to *p* < 0.05

The analysis was conducted to investigate concentration effects of GTM treatment on Rab proteins involved in vesicular trafficking and associated with caveolae. The concentration of four Rab proteins expression varied significantly upon GTM treatment. Rab 8a, Rab13 and Rab3gap2 significantly decreased at GTM concentration of 5 mg/ml; Rab9a significantly increased at 1 mg/ml GTM concentration (Fig. 7).

The Uniprot database lists Rab8a activity in polarized vesicular trafficking, autophagy and neurotransmitter release, furthermore Rab8 activity has been described in transcytosis, in ciliogenesis and in the regulation of adherent junctions assembly [40]. Interestingly Rab8 has been described in two studies involving in hearing loss. The first [41, 42], has been recognized as a binding partner of otoferlin a protein found in ribbon synapses, sensory hair cells and neurons in the cochlea as well as co-localizing with endosomal and Golgi proteins. Mutation in OTOF encoding otoferlin leads to a nonsyndromic deafness named autosomal recessive deafness 9 (DFNB9). The second study [43] identifies Rab8 as partner recruited by the BBSome complex of Bardet-Biedel syndrome (BBS) protein family to promote ciliary biogenesis. Mutations in the BBsome complex induces the Bardet-Biedel pleiotropic syndrome characterized, among other pathologies, by acute and chronic otitis media, resulting in conductive hearing loss in early childhood [44].

Rab9a participates in the transport of proteins between the endosomes and the trans-Golgi network [45–47]. This function is of interest given the presence of melanin granules in the spiral ligament fibrocyte and the high concentration of melanine granules in the intermediate cells. Rab9a is required to regulate the process of unconventional alternative autophagy [48, 49] and mitophagy [50]. Rab13 regulates membrane trafficking between the trans-Golgi network and the recycling endosome [51]. It also regulates tight junctions’ activity, endothelial cells angiogenesis and autophagy [52, 53].

The hydrolysis of Rab-GTP is GTPase activating protein (GAP)-assisted and regulates Rab proteins activity in vesicular trafficking. Rab proteins switch between the guanosine diphosphate (GDP)-bound conformation and the GTP-bound conformation. Rab-GTPase activating proteins (RabGAPs) encourage the Rab proteins to hydrolyze the bound GTP then by the action of a second protein interaction partner, a guanine nucleotide exchange factor (GEF), the GDP can be swapped for GTP. [54, 55]. Rab3gap2 is a regulatory subunit specific for the Rab3 subfamily which is involved in exocytosis, in synaptic and non-synaptic vesicular release of neurotransmitters and hormones and in proliferation, migration and differentiation processes. Furthermore the heterodimeric Rab3Gap1 and Rab3gap2 complex has been shown to modulate autophagosomal biogenesis to influence protein aggregation and to affect autophagy at basal and rapamycin-induced conditions [56].

Finally, the protein concentration measurements in the immunoblots for Rab3a, Rab3b, Rab4, Rab5, Rab6a, Rab6b, Rab7, Rab11, Rab13, Rab22a, Rab23 did not reach significance and are shown as supplemental material (Additional files 5 and 6).

### Nonsyndromic hearing loss proteins segregating with caveolae in SL pericytes

In a previous study it has shown that nonsyndromic pathologies related proteins were associated with cholesterol-rich microdomains [25]. Mutated gene products inducing nonsyndromic pathologies have been described in various tissues and cell types in the inner ear. Overall in this study, we found fifteen proteins previously described in nonsyndromic hearing loss pathologies segregating with caveolae in SL pericytes (Table 5).

**Table 5:** Proteins associated with Non-Syndromic Hearing Loss segregating with caveolae in SL pericytes. The table shows proteins implicated in nonsyndromic hearing loss pathologies segregating with caveolae in treated and untreated cells. The highest number of unique peptides identifying the proteins is given in the table (n CTRL and n GTM) as well as their UniProt identifiers. The proteins myosin heavy chain 14 (MYH14), myosin heavy chain 9 (MYH9), Wolframin (WFS1), Lysyl-tRNA synthase (KARS) have been previously described in the SL. The proteins Diaphanous 1 (DIAPH1), MYH14, MYH9, unconventional myosin VI (MYO6), Radixin (RXD), TRIO and filamentous actin binding protein (TRIOBP), Taperin (TPRN), WFS1, KARS, Serpin B6 (SERPINB6), tight junction protein ZO-2 (Tjp2), polyribonucleotide-nucleotidyl transferase (PNP1), segregated with caveolae in both in untreated and GTM treated cells. One protein, Calcium integrin-binding family member 2 protein (CIB2), exclusively segregated with caveoale in untreated cells and two proteins Methionine Sulfoxide Reductase B3 (MSRB3) and Coiled Coil Domain Containing protein 50 (CCDC50) segregated exclusively in GTM treated cells

Protein name	Gene	Function	n CTRL	n GTM	UniProt ID
Diaphanous 1	DIAPH1	Cytoskeleton and mobility	4	5	O08808
Myosin Heavy Chain14	MYH14	Motor protein	2	2	Q6URW6
Myosin Heavy Chain9	MYH9	Motor protein	100	108	Q8VDD5
Unconventional myosin 6	MYO6	Motor protein	6	11	Q64331
Radixin	RDX	Cytoskeleton	6	10	P26043
TRIO filamentous actin binding protein	TRIOBP	Cytoskeleton	12	12	Q99KW3
Taperin	TPRN	Cytoskeleton	6	6	A2AI08
Wolframin	WFS1	Regulation of Ca^2+^ homeostasis	14	14	P56695
Lysyl-tRNA-synthase	KARS	Attach amino-acid to its cognate tRNA	9	9	Q99MN1
Serpin B6	SERPINB6	Protease inhibitor	10	11	Q60854
Tight junction protein ZO-2	TJP2	Role in tight and adherents junctions	3	3	Q9Z0U1
Polyribonucleotide-nucleotidyl transferase	PNPT1	Degrades mRNA	2	1	Q8K1R3
Calcium integrin binding protein 2	CIB2	Calcium-binding regulatory protein	1		Q9Z309
Methionine R sulfoxide reductase	MSRB3	Antioxidant		2	Q8BU85
Coiled coil domain containing protein	CCDC50	Cytoskeleton		2	Q810U5

Four of these proteins MYH14, MYH9, WFS1 and KARS have been previously described in the SL. MYH14 is a motor protein with poorly understood functions while the MYH9 protein plays a role in cytokinesis, cytoskeleton reorganization and focal contact formation. WFS1 encode for a protein participating in the regulation of cellular Ca^2+^ homeostasis. Finally, KARS derived protein is known to interact with laminin receptor on the cell surface and catalyze specific attachment of amino-acid to its cognate tRNA [57]. The remaining eleven proteins were identified for the first time in SL pericytes. Eight proteins were found expressed both in controls and GTM exposed cells. The group comprises RXD, TRIOBP, MYO6, SERPINB6, Tjp2, DIAPH1, PNP1 and TPRN. One protein, CIB2, was found to be exclusively expressed in control SL pericytes and two proteins, MSRB3 and CCDC50, were found exclusively in GTM exposed cells. CCDC50 encoded protein is involved in epidermal growth factor receptor (EGFR) signaling, while MSRB3 is an antioxidant enzyme that catalyzes the reduction of free and protein-bound methionine sulfoxide to methionine. MSRB3 is an essential protein for hearing since it has been shown that its ablation in MSRB3^−/−^ mice cause profound hearing loss without other pathological symptoms [58].

## Discussion

Crossing the BLB is necessary for GTM to penetrate any cells of the inner ear [8–11]. Delivery across the BLB is also a potential route for therapeutic intervention in order to prevent damages induced by ototoxic drugs and noise. In view of these considerations, SL pericytes and caveolae isolated from these cells provide and effective and appropriate model to investigate changes in protein transport induced by GTM and/or noise. SL pericytes maintain the BLB in the lateral wall [8] of the inner ear. Microvessels in this region have the highest ratio of pericyte/endothelial cell of any microvascular bed in the body [38]; they control the blood flow that is necessary for the maintenance of inner ear perilymph and endolymph [14, 15], whose chemical composition is essential for hearing. SL pericytes are very similar to brain pericytes, which have been shown to control endothelial cells’ transcytosis [16], one of the important functions of caveolae. Caveolae modulate cellular traffic to many cell compartments, from the plasma membrane to endosomes to organelles [17–19] and this makes caveolae and the proteins associated with them important for investigating protein transport in pathological conditions. Acoustic trauma and GTM have been shown to damage the BLB [11, 12]. Our findings using this model show that GTM induces significant changes in the network of caveolae associated Rab transport proteins, with potential impact on BLB physiology and hearing loss.

Indeed our results demonstrate that GTM exposure altered SL pericytes proteomic profile in caveolae. The finding of proteins uniquely segregating with caveolae and the finding of a specific pattern of Rab proteins association with caveolae during the GTM exposure represents a formidable target for therapeutic intervention to the hardly accessible cochlear tissues.

This study shows that 40% of total proteins segregating with caveolae are specifically expressed during GTM exposure, and 15% of the proteins normally expressed in controls are suppressed in cells incubated with GTM. Overall the enrichment analysis showed that GO categories containing proteins participating in energy metabolism regulation, cytoplasmic transport, cell survival, apoptosis, protein synthesis and degradation were significantly overrepresented in the GTM exposed cells while GO categories including proteins participating in genetic information were suppressed during the exposure. Transport-specialized Rab proteins were significantly enriched in the GTM exposed cells.

Immunobloting analysis revealed a significant change in Rab8a, Rab9a, Rab13, and Rab3gap2 protein concentration in treated cells, suggesting a possible change in cellular cargo transport under GTM treatment. Understanding the strong cooperation of caveolae and Rab proteins in cytoplasmic trafficking and the trafficking direction of caveolae cargoes away from degradation or recycling inside the cells is important for successful drug delivery to the inner ear.

Finally, we report here, a specific pattern of proteins implicated in non syndromic pathologies segregated with caveoale. Interestingly a protein essential for hearing (MSRB3) is segregating with caveolae only in the GTM exposed cells, underlining the importance of the antioxidant defense in the inner ear and opening to the possibility for therapeutic intervention through caveolae.

Caveolins had been identified as relevant in a non syndromic pathology linked to hearing loss. Mutations in GJB2, which encodes connexin26, a cochlear gap junction, cause pre-lingual nonsyndromic deafness. Abnormal accumulation of cav1-rich, and cav2-rich vesicles at the gap junctions level, characterized by increased endocytosis and junction disruption, was considered the underlying cause of the pathology [27]. Increased cav2 accumulation in GJB2 mutant mice has been associated with abnormal morphology of the outer hair cells in the organ of Corti. The increased cav2 level contributed dramatically to the progression of the GJB2 –associated deafness [59]. Rab proteins have also been linked to other proteins involved in nonsyndromic deafness. Rab 8b has been recognized as a binding partner of otoferlin, a member of the ferlin family transmembrane anchored proteins whose mutations cause nonsyndromic deafness due to defective neurotransmission. Ferlins of the type-II sub-family, which include otoferlin, localize in the trans-Golgi/recycling network. One member of the sub-family colocalizes with cav3 in endosomes of mouse and human myoblasts [60].

GTM offers an excellent model for studying hearing loss induced by drugs and noise, since both causes have in free radicals, one of the major initiators. Drug-induced and noise-induced hearing losses are the leading causes of hearing impairment and deafness in the world population. The molecular mechanism leading to ototoxicity is not fully elucidated, but reactive oxygen species ROS have been recognized as one of the major culprits. GTM and the related aminoglycoside antibiotics chelate iron, and the resulting iron-aminoglycoside complex is redox-active, catalyzing the formation of ROS [61]. Moreover, increased oxidative stress is associated with both continuous and impulse noise-induced hearing impairment [62, 63]. ROS are considered one of the main culprits for noise-induced hearing loss and deafness. Scientific evidence accumulated since the 1990s shows the appearance of increased ROS and other toxic free radicals, such as superoxide O_2_^-·^ or lipid peroxides, during and after noise exposure [64]. Antioxidants and iron chelators have been shown to protect against both GTM-induced and noise-induced hearing loss. Administration of alpha lipoic acid, a powerful antioxidant and iron chelator, decreases aminoglycoside induced hearing loss in vitro and in vivo [65, 66]. In human subjects, pretreatment with alpha lipoic acid has been shown to protect from prolonged acoustic trauma [67]. Moreover, treatment with the antioxidant N-acetyl–cysteine (NAC), a glutathione precursor and an antioxidant, and the free radical scavenger agent disodium 2,4-disulfophenyl-N-tert-butylnitrone (HPN-07), effectively reduced hearing loss and cochlear hair cell death in rats, when administered after blast exposure [63]. NAC has been shown to reduce noise-induced hearing loss in animal models exposed to continuous noise [68, 69] to protect human subjects from noise-induced cochlear injury in clinical trials [70, 71] and to improve GTM-induced ototoxicity in hemodialysis patients [72]. SL pericytes are key cells for studying the damage induced by aminoglycosides and by noise in the cochlear microvasculature. Synergy and signaling between endothelial cells and pericytes is fundamental for the maintenance of the blood labyrinth barrier BLB [8]. A damaged BLB may be an entrance route for ototoxic drugs. Hypotheses have been raised about vascular aminoglycoside trafficking through the spiral ligament microvasculature and tissue, reaching the endolymph and ultimately the cochlear hair cells [73]. Leaking capillaries and loss of strong association of pericytes with endothelial cells have been shown in the SV of animals exposed to continuous acoustic trauma [74]. SL pericytes encircle endothelial cells in the microvasculature of the SL in a 1:1 to 1:2 ratio, which is similar only to the micro-vessels of the retina 1:1, and the brain 1:5; this is an extremely high ratio compared to most other organs such as the lungs 1:10 and the skeletal muscle 1:100 [38]. The intimate contact with the endothelial cells in the spiral ligament microvasculature, suggests a central role for SL pericytes as a bridge between the blood flow, the endothelial cell and the inner ear tissues, suggesting the possibility of intense signaling and metabolite exchange between the two cell types. An important role for SL pericytes is the control of the blood flow in the inner ear microvasculature. SL pericytes contrary to the stria vascularis pericytes, express contractile proteins such as α-SMA, and have shown contractility in response to stimuli in vitro and in vivo [14, 38]. The ability to contract allows the SL pericytes to regulate the blood flow of the more distal downstream capillaries leading to the SV, [14], This is a critical feature for generating the endocochlear potential, which is indispensable for normal hearing [15]. Pericytes participate in transport across microvasculature, although the role of pericytes in transport and transcytosis in the BLB has been minimally investigated. Early reports described SL pericytes and the BLB sharing similarities with the brain pericytes and with the BBB respectively [75]. A recently published work reported the importance of pericytes in the critical control of endothelial cell transcytosis in the brain microvasculature. The Mfsd2a (major-facilitator superfamily domain-containing-2a) protein-encoding gene is selectively expressed in the BBB microvessels. The genetic ablation of Mfsd2a dramatically increases endothelial cell vesicular transcytosis in KO mice, and the transcytosis in endothelial cells, is controlled by pericytes, through the modulation of Mfsd2a expression [16]. Because of the similarity of the spiral ligament capillaries to the BBB capillaries, it is plausible to speculate a similar regulatory role for SL pericytes in the control of endothelial cells transcytosis in the BLB. Understanding vesicle transport and transcytosis in SL pericytes can shed light on metabolite uptake and transport through inner ear microvasculature in physiological and pathological situations.

Transcytosis and endocytosis are some of the functional roles of caveolae, specialized and morphologically distinct, cholesterol sphingolipids and protein-rich microdomains, stabilized by caveolin proteins [76]. Caveolae are an entrance point to the cell for albumin [77], bacteria, viruses [78], DNA-linked peptides and cross-linked polymeric micelles for drug delivery therapy [79, 80]. Caveolae are present on the plasma membrane as flask shaped microdomains. They can assume complex multi-lobed configurations with tubules linking these assemblies to the cell membrane [81] or exist as vesicles detached from the plasma membrane associated in grape like clusters [82]. Caveolae are also dynamic structures that have been shown to fuse with early endosome and to form caveosome, late endosome and multivesicular bodies [81]. Intense caveolae trafficking occurs underneath the plasma membrane in rapid “kiss and run” cycles in which the caveolar coat stays intact and sequesters multivalent sphingolipids bound cargos [83]. Caveolins, a family of hairpin-like palmitoylated integral membrane proteins that oligomerize and bind to cholesterol and sphingolipids identify caveolae. Cav1 and cav2 are ubiquitously expressed, while cav3 expression is restricted to muscle cells. Cav1 serves as a selective marker for caveolae. Cav1 has an unusual high affinity with cholesterol and resists dissociation even with harsh detergents. Cav1 forms oligomeric complexes in the presence of cholesterol contributing to caveolae genesis [82]. Metabolic depletion of cholesterol or removal of cholesterol from membrane disrupts caveolae [84], as does genetic ablation of cav1 [81]. The unusual lipid composition of caveolae confers buoyancy, resistance to solubilization by non-ionic detergents such as Triton-X-100 at 4 °C. This property together with the marker cav1 and the distinctive buoyancy, form the basis for caveolae characterization, identification and purification.

In this study the caveolae proteins cav1 and cav2 were not depleted in the SL pericytes during the GTM challenge, showing that GTM did not affect the structural integrity of the caveolar microdomain. The complexity and the dynamism of caveolae interactions in the cells physiology is made evident by the thousands of proteins associated with caveolae and is revealed by the mass spectrometry analysis. The differences in the GO terms enriched in the specifically expressed proteins in the GTM and control dataset show the response of the cell in physiological and pathological conditions. The subsequent analysis of proteins isolated from caveolae with bioinformatics tools revealed important patterns in the overrepresented cellular components and processes.

The gene ontology enrichment analysis of the GTM dataset shows that caveolae activity was significantly found in the cytoplasm and in the cell membranes including vacuoles and vesicles, membrane protein complexes, exosome and mitochondria. Within the “Biological process” ontology the enriched GO categories showed significance for the terms localization and transport which show that caveolae actively participate in movement and transport of proteins, lipids and small molecules in the processes and pathways enriched in the analysis.

Transport and localization to membranes and cytoplasmic component has been described in literature and are known interactions and activities established by caveolae in the cell. Caveolae exist as individual microdomains clustering in stable multi-caveolar assemblies or undergoing continuous cycling of fusion and internalization while trafficking to and from the cell membrane, intracellular vesicles and cytoplasm [83]. Interestingly, overrepresented GO categories within the “cellular component” ontology included “Extracellular exosome” and “Mitochondrion”.

The activity of caveolae and cav1 in exosomes has been only recently brought to attention. Exosomes expressing CD63 and cav1 have been described in large amount in plasma of melanoma patients [85]. Caveolae have been shown to participate in uptake and internalization, via endocytosis pathways, of exosomes released from Epstein-Barr virus infected B cells by epithelial cells [86]. Furthermore dynamin, a component of the caveolae endocytic pathway, participates in the uptake of exosomes containing anthrax toxin [87]. Exosomes are potent mediators of intercellular communication; the enrichment of the GO category “extracellular exosome” suggests the existence of pathway(s) utilizing caveolae transport for shuttling exosomes loaded with extracellular material in and out of the cell in response to stress during the GTM challenge. Particularly during pathological conditions extracellular material and plasma membrane proteins are delivered to lysosomes for degradation. Caveolae and exosome cooperation may serve as a cellular mechanism to enhance protein degradation or removal of damaged cellular material, through their release to cellular autophagocytic machinery or to the extracellular environment as exosomes. Immuno-staining and transmission electron microscopy, have recently show that caveolae and cav1 are expressed in mitochondria of rat pulmonary arterial smooth muscle cells [88], and in mitochondria-associated membranes (MEMs) in hepatocytes, connecting the endoplasmic reticulum to mitochondria [89]. Cellular protection from damage induced by a variety of insults has been suggested for the transferring of cav1 between caveolae and mitochondria. Furthermore, it has been shown that the caveolae-mitochondria interaction regulates the adaptation to cellular stress by modulating the structure and function of mitochondria [19] and that cav1 is relevant for mitochondrial functioning and lipids and metabolic homeostasis [55]. GTM is known to induce cell disruption and apoptosis in the inner ear increasing ROS, inducing mitochondria oxidative damage and, consequently, hearing loss. The enrichment of the GO category “Mitochondrion”, and the association with caveolae, shows an important synergy between mitochondria and caveolae in GTM induced stress. This suggests the existence of protective mechanisms in place for cell survival and for mitochondria repair and adaptation to stress, in which caveolae and caveolins play an important role as lipids and molecule transporters to and from the mitochondria, in order either to maintain the energy metabolism or to respond to the increased energy demand of the cell.

The enrichments and the visualization analysis in control and GTM uniquely segregating proteins showed dramatic changes in quite different categories, giving for the first time an insight to the complex proteomic alterations associated with the administration of GTM to cells of the inner ear. The bioinformatic data for the GTM proteome show the cells in an active and stressed status where the significant effort seems to reside in energy boosting, protein degradation, biosynthesis, and vesicular transport. The increase of cell metabolic activity, increased amino acid, fatty acid and nucleotide biosynthesis, upregulated glycolysis, purine metabolism, tricarboxylic acid (TCA) cycle, have been described as a consequence of GTM in small colony variants of *Staphylococcus aureus* [90]. Those same processes are observed in our data (Fig. 5, Table 1A, and Additional file 4). In particular, the TCA cycle is essential for a majority of metabolic pathways and its serves as node connecting catabolic energy gaining pathways with anabolic pathways. Its upregulation provides more biosynthetic intermediates and more redox potential in order to gain energy via oxidative phosphorylation [90] Interestingly, the category oxidative phosphorylation has been shown in our data only in the proteome of GTM challenged cells (Fig. 5, and Additional file 4), demonstrating the need for increased energy possibly to counteract the damage of the drug. The bioinformatic data for the control proteome (Fig. 5, Additional file 4, Tables 3A, B and C and Additional file 3) show that in the healthy cell the caveolae participate in genetic information processing, in transcription, in nucleotides binding and spliceosome activity, in signal transduction, involving nucleus, ribosome, RNA transport, processing and binding, and DNA maintenance. The enriched categories for the cellular component ontology show that nucleic part and nucleus are the most significant, suggesting a novel role for caveolae in transferring and transport of genetic material. Our findings support recent works that contribute to insights for possible alternative of trafficking patterns involving caveolae. Polyplexes formed by polymer-DNA complexes, have been shown to reach the nucleus through a route involving caveolae, in which the polyplexes accumulate into Rab6- labelled Golgi and ER vesicles and ultimately are transported into the nucleus, during ER mediated nuclear envelope reassembly [91]. Moreover, it has been shown in ovarian carcinoma cells that cav1 associates with the nuclear matrix and with inactive chromatin and colocalize with nuclear inner membrane proteins and promoter sequences participating cell cycle progression, suggesting that cav1 exerts a functional activity mediated by binding to sequences of genes involved in proliferation [18]. The difference in the proteomes of physiological and pathological cell is an attractive target for developing therapies for GTM and noise induced hearing loss. Changes in gene products expression, have been reported in a study using RNA sequencing to evaluate the response of inner ear hair cells to GTM. The study showed that within 3 h of GTM treatment, the mRNA level of more than three thousand genes in the hair cells changed significantly [92]. Moreover, mice exposed to repeated acoustic blasts showed injury to the auditory cortex and significant alterations in the expression of multiple genes in the brain, known to be involved in age- or noise-induced hearing impairment [93].

The specifically expressed proteins dataset from GTM treated cells showed that the highest enrichment significance was reached in the GO category “Transferase activity transferring phosphorus”. Proteins in this category encompass all kinase activities participating in the transfer of a phosphorous containing group from one donor to an acceptor, thus activating pathways and cellular processes. The proteins listed in the category are mostly protein kinases and in particular protein serine/threonine kinases. Interestingly, the dynamic properties of caveolae and their transport competence are regulated by kinases. In particular, it has been shown that serine/threonine kinases differentially regulate caveolae short and long distance cycling between the cell surface and intracellular organelles, and that caveolae move more upon kinase activation [83]. A molecular switch triggered by kinase activation suggests the increase of caveolae dynamism in GTM treated cells. Caveolae increased transport activity is confirmed in the data of the GOrilla enrichment analysis and is visualized in the Proteomaps, where “Transport” became the dominant subcategory in the top area “Cellular process”. A change of the cycling mode from short to long range, and increased caveolae transport activity and kinase activation are then plausible consequences of GTM administration to the cells.

The identification of proteins uniquely segregating with caveolae during the GTM challenge in the inner ear microvasculature pericytes is formidable knowledge, since those proteins are likely to be expressed predominantly or specifically when the inner ear undergoes ototoxic or noise-induced damage. These proteins are attractive targets, reachable with therapeutic vectors through the bloodstream to the hardly accessible inner ear.

The caveolae-mediated endocytosis process would offer a significant advantage for delivering its load across the cells efficiently, enhancing deep tissue and vascular penetration of its cargo drugs. Activation of the caveolae endocytic pathway has been shown in studies investigating the internalization of co-polymers as potential drug delivery vehicles [94].

Transport functions are controlled by Rab proteins, which are key regulators of intracellular membrane trafficking. Activated Rabs switch from a GDP form to a GTP form. Interestingly, phosphate-transferring group was a category enriched in the GTM uniquely expressed proteins suggesting the activation of Rab pathways. Remarkably, only Rab proteins in the GTM dataset and not in control cells were significantly enriched, demonstrating the relevance of the Rab-caveolae cooperation in pathological condition. The increased segregation of Rab proteins with caveolae in GTM treated cells and the significant enrichment of the pool of Rab expressed in the GTM treated vs untreated cells could be considered as a marker of cellular stress for pathological condition such as the GTM administration.

Once activated, Rabs are able to recruit (to different membranes) different sets of downstream effectors responsible for all steps of vesicle trafficking [95] which include regulation of vesicular movements, endocytosis, movements of vesicles packaged with cargo proteins, association with motor proteins that facilitate the movement of vesicles along actin, microtubules, cytoskeleton tethering factors that dock vesicles to target compartments for membrane fusion. Each distinct membrane compartment is targeted by a particular set of Rab proteins. Evidence suggests that the Rabs and caveolae share a common path in the cell transport processes. Budded caveolae, can fuse with the early endosome in a Rab5-dependent manner [20]. In HeLa cells, cholera toxin and simian virus 40 (SV40) are transported to endosomes by caveolar vesicles in a process dependent on Rab5 [96]. The coordinated interactions of cav1 and Rab11 regulate the apical recycling compartments in polarized epithelial cells [21]. In glial cells the human polyomavirus JC virus is sorted from early endosome to a cav1 positive endosomal compartment in a Rab5 dependent step [97]. Overexpression of Rab7 and Rab9 in Niemann-Pick C fibrocytess mediate the Golgi transport of caveolae-internalized glycosphingolipids reducing intracellular accumulation of cholesterol and increasing accumulation of neutral lipids [98]. The specificity of Rab transport in hearing loss is slowly gaining interest and is still rather scattered. In a study assessing gene expression in guinea pig paraflocculus structure in the cerebellum after acoustic trauma, the protein Rab3A, which is involved in regulation of pre-synaptic neurotransmitter release, showed no changes [99]. A Mutation in the gene TBC1 Domain Family Member 24 (TBC1D24), encoding for a GTPase activating the proteins Rab5 and Rab35, involved in endosome to lysosome trafficking as a mechanism for degrading synaptic vesicles associated proteins, has been shown to cause severe neurodegeneration and deafness [100]. In HEK 293 cells otoferlin, a protein involved in human autosomal recessive deafness, is co-localized and co-expressed with Rab8 contributing with trans-Golgi trafficking [41]. In a recently published study, our group showed that a Single Nucleotide Polymorphism (SNP) of the Rab11 effector Rab11Fip1 is associated with a noise-induced hearing loss [101]. In this study, cells incubated with GTM for 24 h at 1 mg/ml and 5 mg/ml significantly modified the concentration of some of the Rab proteins tested. The significant change in Rab8a, Rab9a, Rab13, Rab3gap2 shows the concentration effect of GTM on a family of transport proteins, that interacts with multiple cell compartments and may influence more significant changes in the cells. A concept recently emerging in Rab biology suggests that compartment conversion in endosome maturation is induced through a change in Rabs and their effector molecules, that ultimately alters membrane function and identity [102], and may affect the directional flow of cell cargo transport in the cell.

Rab8a, Rab9a, Rab13, Rab3gap2 share activity in several pathways. All four Rabs play a role in autophagy. Rab 9a and Rab13 are active in vesicular protein transport through the trans-Golgi network to endosomes. Rab8a and Rab3gap2 share activities in extracellular vesicular transport. Rab8a and Rab13 play a role in cell-cell junctions and in the transport of the glucose transporter protein GLUT4, which regulates the glucose [103].

Autophagy is a protective process activated in the cells to adapt to various stresses. The fact that four Rabs in a pool of Rab proteins isolated with caveolae shared significance in autophagy pathways is of particular interest. Recent studies have shown the participation of cav1 in autophagy process, in particular it has been shown that phosphorylated cav1 functions to activate autophagy under several stress conditions [104].

Rab8a and Rab13 share trans-Golgi network vesicular transport activity. The trans-Golgi network is a major sorting, packing and delivering station of newly synthesized proteins and lipids to their destinations [105] The significant changes of Rab9a and Rab13 following the GTM treatment may suggest impairment to lipids and proteins transport, packing and sorting potentially contributing to changes in the cells vesicular membranes and transport pathways.

Extracellular cargo transport may be affected by the GTM incubation. Rab8a and Rab3gap2 described respectively in transcytosis and exocytosis significantly decreased at the 5 mg/ml concentration. Transcytosis is one of the potential roles described for caveolae. Numerous findings propose that caveolae transcytose low density lipoporteins (LDLs), albumin and insulin [106]. Rab8a has been associated with basolateral-to-apical transcytosis in polarized Madin-Darby Canine Kidney Cells (MDCK) cells [107], while Rab3gap2 had been shown to form with the protein Rab3gap1 a complex acting in exocytosis and endocytosis, and extracellular trafficking [108].

Two of the Rabs tested, Rab8a and Rab13, showed a significant decrease after GTM incubation. Both Rabs have been shown to play a role in cell-cell junctions. Rab8a has been described in adherens junctions [109], while Rab13 has been described in tight junctions [110]. Pericytes on the vessels of the SL strongly expressed gap junction proteins [38], and tight junctions are present in the cells of the lateral wall and BLB. [111]. Furthermore, caveolae and cav1 have been associated with tight junction organization in the cerebral endothelium [112] and have been shown to impact blood brain permeability in ischemia reperfusion injury [113]. Finally, the insulin regulated glucose transporter GLUT4 localizes to caveolae after translocation to the plasma membrane [114]. Glucose deprivation in pheochromocytoma (PC)12 cells translocates GLUT4 to the cell membrane, up-regulates both cav-1 and GLUT4 and changes mitochondrial membrane potential [115]. Taken together these findings underline the importance of understanding the intracellular trafficking machinery that associate Rabs and Caveolae for any manipulation of caveolae and their cargo in the cytoplasm.

## Conclusion

The results shown in the study demonstrate that GTM exposure of SL pericytes induces changes in caveolae proteome profile, specifically and significantly modifying the expression of protein-encoding genes during the challenge. Furthermore, the changes in protein expression impact the transport Rab GTPases, significantly over representing pathways leading to the cell proteolytic machinery, exocytosis, cytoplasm to membrane transport and recycling and transport to and from the Golgi apparatus. Finally, we describe for the first time proteins associated with nonsyndromic deafness in SL pericytes.

Our findings show that about 40% of the proteins segregating with caveolae were uniquely found in the cells challenged with GTM. These results are interesting in view of the caveolae localization on the cell membrane, its endocytic and transcytotic activity in the cell cytoplasm and the possibility of exploiting these features for drug delivery to the hardly accessible cochlear inner ear. Specifically expressed proteins could constitute a target site for docking systemically administered blood-borne vectors, carrying therapeutic agents, to be delivered to the cochlear tissues. Insights and understanding of Rab vesicular transport routes in the cell cytoplasm during cochlear damage would allow the manipulation of caveolae cytoplasmic path, to precisely and selectively direct the caveolae and their cargoes.

## Additional files

Additional file 1: Caveolin-1 Dot Blot analysis of gradient aliquots. Caveolae-rich aliquots from CTRL and GTM treated cell lysates. Optiseal gradients previously loaded with cell lysates were fractionated in 8 to 9 aliquots after the ultracentrifugation. Cav-1 signal was obtained with Dot-Blot on PVDF membrane using 3 μl from each gradient aliquot using anti-cav-1 antibody (Sigma-Aldrich, USA) with overnight incubation. The aliquots with the strongest signal for cav-1 were selected for protein separation and mass spectrometry analysis. The blots are representative of three independent experiments. (PPTX 251 kb)

Additional file 2: Venn diagram from the three mass spectrometry experiments. Only proteins detected at least in two of the three mass spectrometry runs were used to build the diagram and further used in the bioinformatics analysis. One thousand six hundred eighty two proteins were found in the control set and 2379 proteins in the GTM set. Among these, 948 proteins (40%) were uniquely segregating with caveolae in GTM-treated cells; 251 proteins (15%) were uniquely segregating with caveolae in the control dataset and 1431 proteins were commonly expressed. (PPTX 106 kb)

Additional file 3: **Table S3** A, B and C. Enrichment analysis of proteins uniquely segregating with caveolae in untreated cells. The 251 proteins uniquely segregating with caveolae in untreated cells where selected as the target group for the GOrilla enrichment analysis. The control dataset plus the GTM dataset were chosen as background group. The table shows the complete list of significantly enriched GO terms to FDR q-value< 0.05. The enrichment showed significance for terms in the categories “Biological process”, “Cellular component” and “Molecular function”. The enriched terms showed the suppressed activities and functions in the cells once GTM is administered. (DOCX 34 kb)

Additional file 4: Proteomaps of the proteins uniquely segregating with caveolae and untreated cells. Comparative visualization of the proteins uniquely segregating with caveolae in control and GTM treated cells**.** The two panels show the further division of the top area polygons (see Fig. 5) in sub-categories for the control and the GTM dataset respectively. (TIFF 6509 kb)

Additional file 5: Rabs immunoblotting. SL pericytes were incubated with increasing concentrations of GTM (1 mg/ml, 5 mg/ml,10 mg/ml GTM) for 24 h. Immunoblots were obtained for each Rab protein from the whole cell lysate. Protein quantification is expressed as the relative quantity to the control for each Rab. Each graph is the result of *n* = 5 independent experiments for Rab3a, Rab4, Rab5, Rab22; *n* = 4 independent experiments for Rab6b, Rab7, Rab23; *n* = 3 independent experiments for Rab11 and Rab3b; *n* = 2 independent experiments for Rab6a. SEM was calculated for each group. Although not significant, a trend can be drawn from the analysis. Three Rab proteins Rab3a, Rab 6a, Rab7 showed a slight increase at 5 mg/ml. Rab11 and 22a showed no change at any concentration tested. In the Fig. A = Rab3a; B = Rab3b; C = Rab4; D = Rab 6a; E = Rab6b; F = Rab5; G = Rab7; H = Rab11; I = Rab23 L = Rab22a. (PPTX 9718 kb)

Additional file 6: Rabs immunoblotting. SL pericytes were incubated with increasing concentrations of GTM (1 mg/ml, 5 mg/ml,10 mg/ml GTM) for 24 h. Immunoblots were obtained for each Rab protein from the whole cell lysate. Protein quantification is expressed as the relative quantity to the control for each Rab. Each graph is the result of n = 5 independent experiments for Rab3a, Rab4, Rab5, Rab22; n = 4 independent experiments for Rab6b, Rab7, Rab23; n = 3 independent experiments for Rab11 and Rab3b; n = 2 independent experiments for Rab6a. SEM was calculated for each group. Although not significant, a trend can be drawn from the analysis. Three Rab proteins Rab3a, Rab 6a, Rab7 showed a slight increase at 5 mg/ml. Rab11 and 22a showed no change at any concentration tested. In the Fig. A = Rab3a; B = Rab3b; C = Rab4; D = Rab 6a; E = Rab6b; F = Rab5; G = Rab7; H = Rab11; I = Rab23 L = Rab22a. (PPTX 7067 kb)

## Abbreviations

AF: Autophagosome
AMPK: Adenosine monophosphate-activated protein kinase
BBB: Blood brain barrier
BBS: Bardet-Biedel syndrome
BBSome: Complex of seven BBS proteins
BLB: Blood labyrinth barrier
CaM: Calmodulin
cAMP: Cyclic adenosine monophosphate
CAV1: Caveolin1
CAV2: Caveolin2
CAV3: Caveolin3
CCDC50: Coiled Coil Domain Containing protein
CIB2: Calcium integrin-binding family member 2 protein
CTRL: Control
CX26: Connexin 26
DFNB1: Nonsyndromic autosomal recessive deafness 1
DFNB9: Nonsyndromic autosomal recessive deafness 9
DIAPH1: Diaphanous 1
DPBS: Dulbecco’s Phosphate-Buffered Saline
EGFR: Epidermal growth factor receptor
FBS: Fetal Bovine Serum
FDR: False discovery rate
GAP: GTPase activating protein
GDP: *Guanosine diphosphate*
GEF: Guanine nucleotide exchange factor
GJB2: Gap junction protein beta 2
GLUT4: Insulin-regulated glucose transporter4
GO: Gene ontology
GOrilla: *Gene Ontology enRIchment anaLysis and visuaLizAtion tool*
GTP: *Guanosine triphosphate*
HEK 293: Human embryonic kidney 293
HeLa: Henrietta Lacks
HPN-07: Disodium 2,4-disulfophenyl-N-tert-butylnitrone
JNK: c-Jun N-terminal kinase
KARS: Lysyl-tRNA synthase
KEGG: Kyoto Encyclopedia of Genes and Genomes
LC MS/MS: Mass spectrometry
MAP: Mitogen-activated protein (kinase)
MDCK: Madin-Darby Canine Kidney Cells
MEMs: Mitochondria-associated membranes
Mfsd2a: Major Facilitator Superfamily Domain Containing 2A
MSRB3: Methionine Sulfoxide Reductase B3
MYH14: Myosin heavy chain 14
MYH9: Myosin heavy chain 9
MYO6: Unconventional myosin VI
NAC: N-acetyl–cysteine
NFkB: Nuclear factor kappa-light-chain-enhancer of activated B
NG2: Chondroitin sulfate proteoglycan 4
PBS-T: Phosphate Buffered Saline with Tween 20
PC12: Pheochromocytoma 12
PH: Phagocytosis
PI: Propidium iodide
PKC: Protein kinase C
PNP1: Polyribonucleotide-nucleotidyl transferase
PVDF: Polyvinylidene fluoride
ROS: Reactive oxygen species
RXD: Radixin
SERPINB6: Serpin B6
SL pericytes: Spiral ligament pericytes
SL: Spiral ligament
SV: Stria vascularis
SVs: Secretory vesicles
T: Transcytosis
TBC1D24: TBC1 Domain Family Member 24
TBST: Tris-buffered saline with Tween
TCA: Tricarboxylic acid
Tjp2: Tight junction protein ZO-2
TPRN: Taperin
TRIOBP: TRIO and filamentous actin binding protein
VEGF: Vascular endothelial growth factor
vWF: von Willebrand factor
WFS1: Wolframin
α-SMA: Alpha-Smooth-Muscle-Actin
