# Supplementary figures and images for: In vitro gentamicin exposure alters caveolae protein profile in cochlear spiral ligament pericytes
Source: Proteome Sci. 2018 Mar 16;16:7. doi: 10.1186/s12953-018-0132-x (PMC5938607; doi:10.1186/s12953-018-0132-x)

## Slide 1
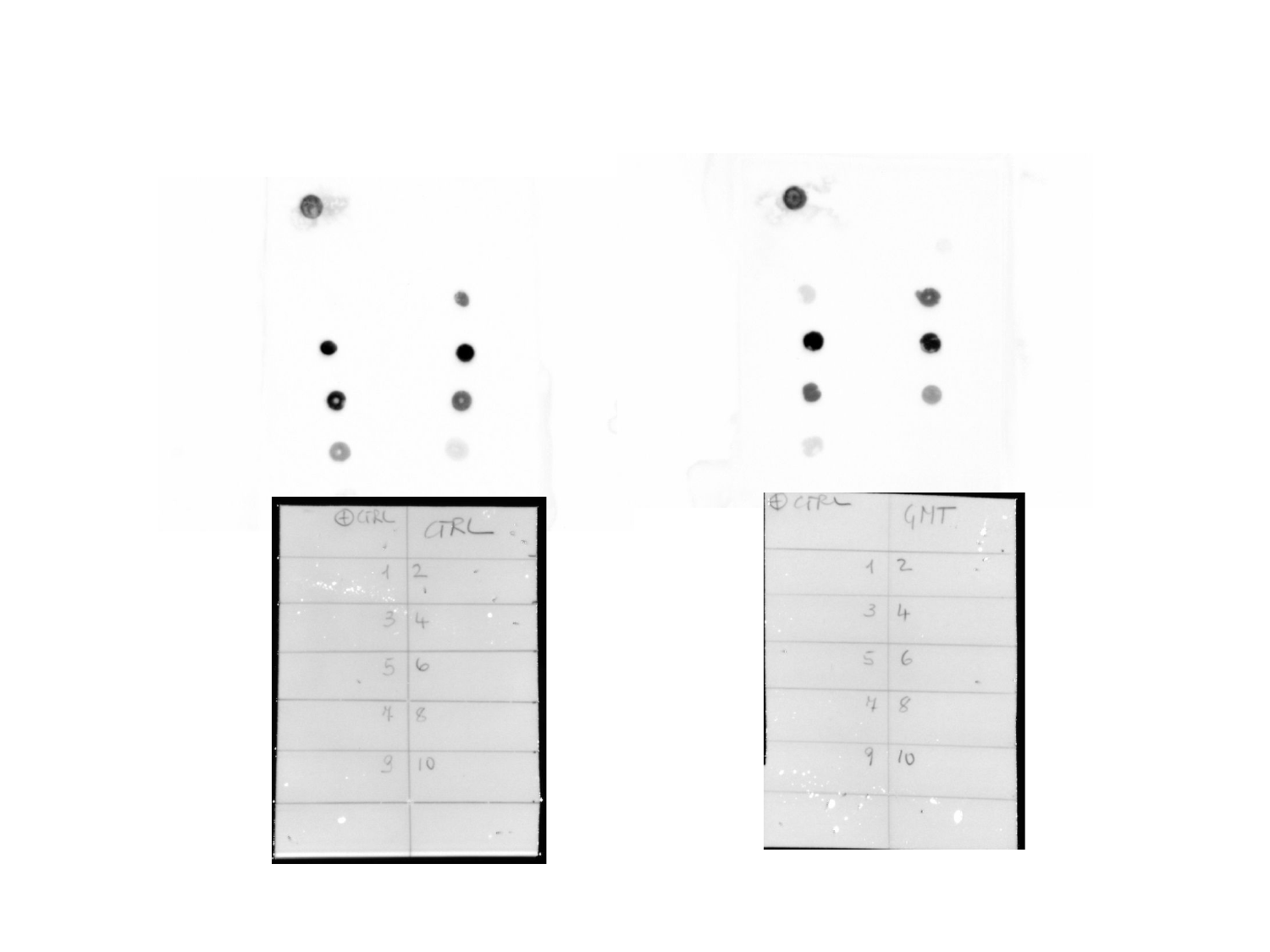

Supplement: Supplementary file 1 — Caveolin-1 Dot Blot analysis of gradient aliquots. Caveolae-rich aliquots from CTRL and GTM treated cell lysates. Optiseal gradients previously loaded with cell lysates were fractionated in 8 to 9 aliquots after the ultracentrifugation. Cav-1 signal was obtained with Dot-Blot on PVDF membrane using 3 μl from each gradient aliquot using anti-cav-1 antibody (Sigma-Aldrich, USA) with overnight incubation. The aliquots with the strongest signal for cav-1 were selected for protein separation and mass spectrometry analysis. The blots are representative of three independent experiments. (PPTX 251 kb) [file 12953_2018_132_MOESM1_ESM.pptx]

## Slide 1
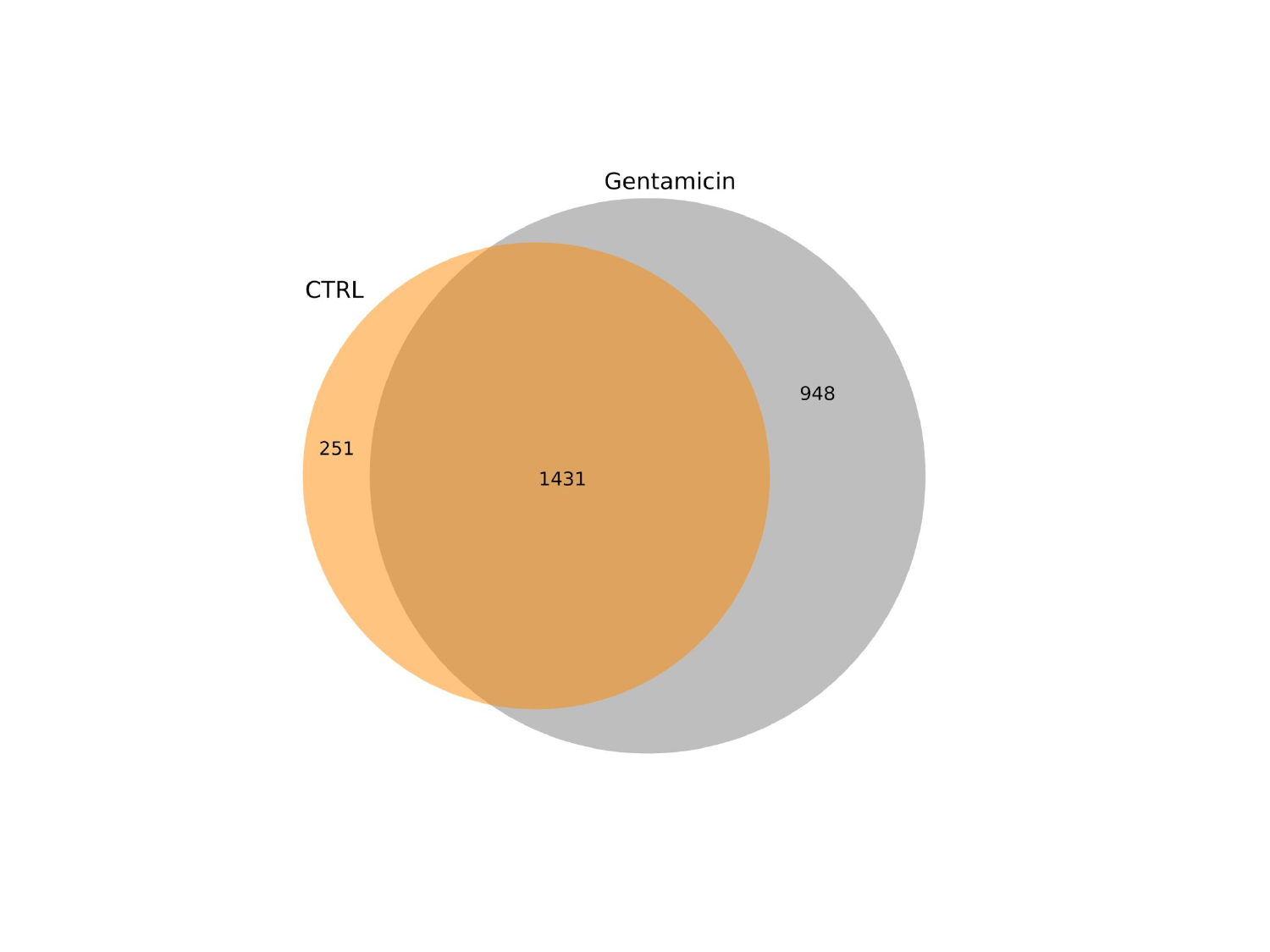

Supplement: Supplementary file 2 — Venn diagram from the three mass spectrometry experiments. Only proteins detected at least in two of the three mass spectrometry runs were used to build the diagram and further used in the bioinformatics analysis. One thousand six hundred eighty two proteins were found in the control set and 2379 proteins in the GTM set. Among these, 948 proteins (40%) were uniquely segregating with caveolae in GTM-treated cells; 251 proteins (15%) were uniquely segregating with caveolae in the control dataset and 1431 proteins were commonly expressed. (PPTX 106 kb) [file 12953_2018_132_MOESM2_ESM.pptx]

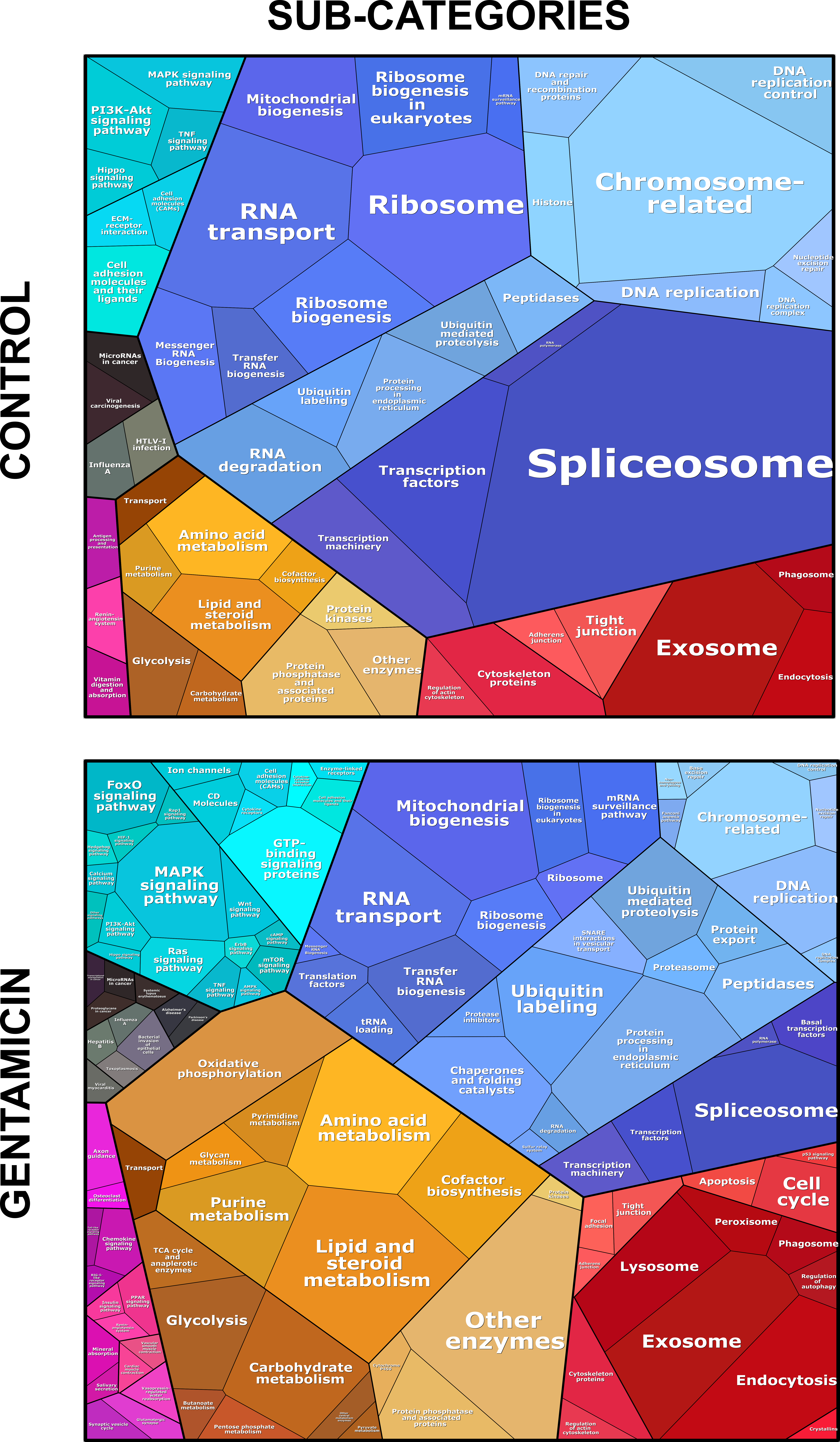

Supplement: Supplementary file 4 — Proteomaps of the proteins uniquely segregating with caveolae and untreated cells. Comparative visualization of the proteins uniquely segregating with caveolae in control and GTM treated cells. The two panels show the further division of the top area polygons (see Fig. 5) in sub-categories for the control and the GTM dataset respectively. (TIFF 6509 kb) [file 12953_2018_132_MOESM4_ESM.tif]
