# Supplementary material for: In vitro gentamicin exposure alters caveolae protein profile in cochlear spiral ligament pericytes
Source: Proteome Sci. 2018 Mar 16;16:7. doi: 10.1186/s12953-018-0132-x (PMC5938607; doi:10.1186/s12953-018-0132-x)
Supplement: Supplementary file 3 — Table S3 A, B and C. Enrichment analysis of proteins uniquely segregating with caveolae in untreated cells. The 251 proteins uniquely segregating with caveolae in untreated cells where selected as the target group for the GOrilla enrichment analysis. The control dataset plus the GTM dataset were chosen as background group. The table shows the complete list of significantly enriched GO terms to FDR q-value< 0.05. The enrichment showed significance for terms in the categories “Biological process”, “Cellular component” and “Molecular function”. The enriched terms showed the suppressed activities and functions in the cells once GTM is administered. (DOCX 34 kb) [file 12953_2018_132_MOESM3_ESM.docx]

| Table 1A. Biological process | | | |  |
| --- | --- | --- | --- | --- |
| GO term | **Description** | **FDR q-value** | **Enrichment (N, B, n, b)** | |
| GO:0090304 | nucleic acid metabolic process | 2.59E-10 | 1.96 (2597,537,249,101) | |
| GO:0016070 | RNA metabolic process | 3.1E-10 | 2.06 (2597,455,249,90) | |
| GO:0006139 | nucleobase-containing compound metabolic process | 1.86E-5 | 1.61 (2597,673,249,104) | |
| GO:0046483 | heterocycle metabolic process | 4.34E-5 | 1.57 (2597,704,249,106) | |
| GO:0006725 | cellular aromatic compound metabolic process | 6.41E-5 | 1.56 (2597,694,249,104) | |
| GO:0006351 | transcription, DNA-templated | 7.04E-5 | 2.23 (2597,210,249,45) | |
| GO:0097659 | nucleic acid-templated transcription | 6.03E-5 | 2.23 (2597,210,249,45) | |
| GO:0010468 | regulation of gene expression | 7.14E-5 | 1.66 (2597,539,249,86) | |
| GO:0032774 | RNA biosynthetic process | 8.61E-5 | 2.19 (2597,214,249,45) | |
| GO:0006396 | RNA processing | 9.71E-5 | 2.13 (2597,230,249,47) | |
| GO:0034641 | cellular nitrogen compound metabolic process | 2.02E-4 | 1.45 (2597,839,249,117) | |
| GO:0008380 | RNA splicing | 2.07E-4 | 2.53 (2597,132,249,32) | |
| GO:0044260 | cellular macromolecule metabolic process | 3.92E-4 | 1.40 (2597,938,249,126) | |
| GO:1901360 | organic cyclic compound metabolic process | 5.68E-4 | 1.58 (2597,561,249,85) | |
| GO:0051171 | regulation of RNA metabolic process | 1.31E-3 | 1.67 (2597,424,249,68) | |
| GO:0051252 | regulation of nucleobase-containing compound metabolic process | 1.49E-3 | 1.60 (2597,495,249,76) | |
| GO:0019219 | regulation of transcription from RNA polymerase II promoter | 1.43E-3 | 2.04 (2597,210,249,41) | |
| GO:0051254 | positive regulation of RNA metabolic process | 1.62E-3 | 2.05 (2597,204,249,40) | |
| GO:0043170 | macromolecule metabolic process | 2.09E-3 | 1.34 (2597,1030,249,132) | |
| GO0006807 | nitrogen compound metabolic process | 2.19E-3 | 1.38 (2597,883,249,117) | |
| GO:0010556 | regulation of macromolecule biosynthetic process | 2.18E-3 | 1.58 (2597,494,249,75) | |
| GO:0016071 | mRNA metabolic process | 2.48E-3 | 2.12 (2597,172,249,35) | |
| GO:0040029 | regulation of gene expression, epigenetic | 2.56E-3 | 4.97 (2597,21,249,10) | |
| GO:0006355 | regulation of transcription, DNA-templated | 2.72E-3 | 1.68 (2597,378,249,61) | |
| GO:1903506 | regulation of nucleic acid-templated transcription | 2.86E-3 | 1.68 (2597,379,249,61) | |
| GO:2000112 | regulation of cellular macromolecule biosynthetic process | 2.99E-3 | 1.58 (2597,483,249,73) | |
| GO:2001141 | regulation of RNA biosynthetic process | 2.9E-3 | 1.67 (2597,380,249,61) | |
| GO:1902680 | positive regulation of RNA biosynthetic process | 3.23E-3 | 2.05 (2597,183,249,36) | |
| GO:0045893 | positive regulation of transcription, DNA-templated | 3.13E-3 | 2.05 (2597,183,249,36) | |
| GO:1903508 | positive regulation of nucleic acid-templated transcription | 3.02E-3 | 2.05 (2597,183,249,36) | |
| GO:0031326 | regulation of cellular biosynthetic process | 3.09E-3 | 1.55 (2597,512,249,76) | |
| GO:0009889 | regulation of biosynthetic process | 4.08E-3 | 1.53 (2597,525,249,77) | |
| GO:0006259 | DNA metabolic process | 4.36E-3 | 2.22 (2597,136,249,29) | |
| GO:0045935 | positive regulation of nucleobase-containing compound metabolic process | 6.6E-3 | 1.82 (2597,252,249,44) | |
| GO:0010628 | positive regulation of gene expression | 7.15E-3 | 1.80 (2597,261,249,45) | |
| GO:0009059 | macromolecule biosynthetic process | 8.85E-3 | 1.60 (2597,405,249,62) | |
| GO:0034645 | cellular macromolecule biosynthetic process | 1.06E-2 | 1.59 (2597,399,249,61) | |
| GO:0006397 | mRNA processing | 1.7E-2 | 2.03 (2597,154,249,30) | |
| GO:0060255 | regulation of macromolecule metabolic process | 1.67E-2 | 1.35 (2597,801,249,104) | |
| GO:0010557 | positive regulation of macromolecule biosynthetic | 1.87E-2 | 1.78 (2597,240,249,41) | |
| GO:0051173 | positive regulation of nitrogen compound metabolic process | 2.1E-2 | 1.70 (2597,282,249,46) | |
| GO:0000122 | negative regulation of transcription from RNA polymerase II promoter | 2.11E-2 | 2.43 (2597,86,249,20) | |
| GO:0080090 | regulation of primary metabolic process | 4.22E-2 | 1.33 (2597,800,249,102) | |
| GO:0045892 | negative regulation of transcription, DNA-templated | 4.94E-2 | 1.96 (2597,149,249,28) | |
| GO:1903507 | negative regulation of nucleic acid-templated transcription | 4.83E-2 | 1.96 (2597,149,249,28) | |
| GO:0051172 | negative regulation of nitrogen compound metabolic process | 4.75E-2 | 1.75 (2597,227,249,38) | |
| GO:0034654 | nucleobase-containing compound biosynthetic process | 4.75E-2 | 1.64 (2597,293,249,46) | |
| GO:0045934 | negative regulation of nucleobase-containing compound metabolic process | 4.96E-2 | 1.81 (2597,196,249,34) | |

| Table 1B. Cellular component | | | |
| --- | --- | --- | --- |
| GO term | **Description** | **FDR q-value** | **Enrichment (N, B, n, b)** |
| GO:0044428 | nuclear part | 1.15E-6 | 1.54 (2597,855,249,126) |
| GO:0005634 | nucleus | 4.66E-6 | 1.38 (2597,1160,249,154) |
| GO:0005681 | spliceosomal complex | 9.9E-4 | 2.66 (2597,98,249,25) |
| GO:0044427 | chromosomal part | 1.89E-3 | 2.01 (2597,208,249,40) |
| GO:00444220 | organelle part | 1.61E-3 | 1.20 (2597,1612,249,186) |
| GO:0044446 | intracellular organelle part | 3.96E-3 | 1.20 (2597,1597,249,183) |
| GO:0000785 | chromatin | 3.41E-3 | 2.50 (2597,96,249,23) |
| GO:1990904 | ribonucleoprotein complex | 4.25E-3 | 1.76 (2597,284,249,48) |
| GO:0030529 | intracellular ribonucleoprotein complex | 3.77E-3 | 1.76 (2597,284,249,48) |
| GO:0043228 | non-membrane-bounded organelle | 5.59E-3 | 1.39 (2597,734,249,98) |
| GO:00432320 | intracellular non-membrane-bounded organelle | 5.08E-3 | 1.39 (2597,734,249,98) |
| GO:0000790 | nuclear chromatin | 5E-3 | 2.93 (2597,57,249,16) |
| GO:0071565 | nBAF complex | 7.68E-3 | 10.43 (2597,4,249,4) |
| GO:0044454 | nuclear chromosome part | 7.98E-3 | 2.19 (2597,119,249,25) |
| GO:0000792 | heterochromatin | 1.17E-2 | 4.08 (2597,23,249,9) |
| GO:0032993 | protein-DNA complex | 2.19E-2 | 4.17 (2597,20,249,8) |
| GO:0071013 | catalytic step 2 spliceosome | 2.14E-2 | 2.76 (2597,53,249,14) |
| GO:0071564 | npBAF complex | 2.56E-2 | 8.34 (2597,5,249,4) |
| GO:0016514 | SWI/SNF complex | 2.43E-2 | 8.34 (2597,5,249,4) |
| GO:0090544 | BAF-type complex | 2.31E-2 | 8.34 (2597,5,249,4) |
| GO:0005694 | chromosome | 4.1E-2 | 2.13 (2597,98,249,20) |

| Table 1C. Molecular function | | | |
| --- | --- | --- | --- |
| GO term | **Description** | **FDR q-value** | **Enrichment (N, B, n, b)** |
| GO:0003676 | nucleic acid binding | 5.68E-10 | 1.70 (2597,779,249,127) |
| GO:0003677 | DNA binding | 1.58E-7 | 2.36 (2597,243,249,55) |
| GO:0000975 | regulatory region DNA binding | 8.02E-7 | 3.56 (2597,79,249,27) |
| GO:0044212 | transcription regulatory region DNA binding | 6.01E-7 | 3.56 (2597,79,249,27) |
| GO:0001067 | regulatory region nucleic acid binding | 9.13E-7 | 3.48 (2597,81,249,27) |
| GO:0043565 | sequence-specific DNA binding | 1.34E-5 | 3.09 (2597,91,249,27) |
| GO:1990837 | sequence-specific double-stranded DNA binding | 4.73E-5 | 3.48 (2597,63,249,21) |
| GO:0000976 | transcription regulat. region seq.-specific DNA binding | 1.24E-4 | 3.54 (2597,56,249,19) |
| GO:0003690 | double-stranded DNA binding | 1.9E-4 | 3.00 (2597,80,249,23) |
| GO:0000977 | RNA polymerase II regulat. region seq.-specific DNA | 8.22E-4 | 3.55 (2597,47,249,16) |
| GO:0001012 | RNA polymerase II regulat. region DNA binding | 7.48E-4 | 3.55 (2597,47,249,16) |
| GO:0097159 | organic cyclic compound binding | 9.41E-4 | 1.30 (2597,1191,249,148) |
| GO:1901363 | heterocyclic compound binding | 9.44E-4 | 1.30 (2597,1182,249,147) |
| GO:0000987 | core promoter proximal region seq.-specific DNA binding | 1.72E-3 | 3.87 (2597,35,249,13) |
| GO:0001159 | core promoter proximal region DNA binding | 1.6E-3 | 3.87 (2597,35,249,13) |
| GO:0001071 | nucleic acid binding transcription factor activity | 2.64E-3 | 2.93 (2597,64,249,18) |
| GO:0003700 | transcription factor activity, seq.-specific DNA binding | 2.48E-3 | 2.93 (2597,64,249,18) |
| GO:0003723 | RNA binding | 3.46E-3 | 1.46 (2597,630,249,88) |
| GO:0003682 | chromatin binding | 3.92E-3 | 2.45 (2597,98,249,23) |
| GO:0044822 | poly(A) RNA binding | 6.35E-3 | 1.49 (2597,531,249,76) |
| GO:0003777 | microtubule motor activity | 1.38E-2 | 6.26 (2597,10,249,6) |
| GO:0000981 | RNA polymerase II transcription factor activity, seq.-specific DNA binding | 1.34E-2 | 3.15 (2597,43,249,13) |
| GO:0000978 | RNA polymerase II core promoter proximal region seq.-specific DNA binding | 1.65E-2 | 3.48 (2597,33,249,11) |
| GO:0031491 | nucleosome binding | 2.07E-2 | 4.39 (2597,19,249,8) |
| GO:0001228 | transcriptional activator activity, RNA polymerase II transcription regulatory region sequence-specific binding | 3.22E-2 | 3.75 (2597,25,249,9) |

Table 3
