# Supplementary material for: In vitro gentamicin exposure alters caveolae protein profile in cochlear spiral ligament pericytes
Source: Proteome Sci. 2018 Mar 16;16:7. doi: 10.1186/s12953-018-0132-x (PMC5938607; doi:10.1186/s12953-018-0132-x)
Supplement: Supplementary file 5 — Rabs immunoblotting. SL pericytes were incubated with increasing concentrations of GTM (1 mg/ml, 5 mg/ml,10 mg/ml GTM) for 24 h. Immunoblots were obtained for each Rab protein from the whole cell lysate. Protein quantification is expressed as the relative quantity to the control for each Rab. Each graph is the result of n = 5 independent experiments for Rab3a, Rab4, Rab5, Rab22; n = 4 independent experiments for Rab6b, Rab7, Rab23; n = 3 independent experiments for Rab11 and Rab3b; n = 2 independent experiments for Rab6a. SEM was calculated for each group. Although not significant, a trend can be drawn from the analysis. Three Rab proteins Rab3a, Rab 6a, Rab7 showed a slight increase at 5 mg/ml. Rab11 and 22a showed no change at any concentration tested. In the Fig. A = Rab3a; B = Rab3b; C = Rab4; D = Rab 6a; E = Rab6b; F = Rab5; G = Rab7; H = Rab11; I = Rab23 L = Rab22a. (PPTX 9718 kb) [file 12953_2018_132_MOESM5_ESM.pptx]

## Slide 1
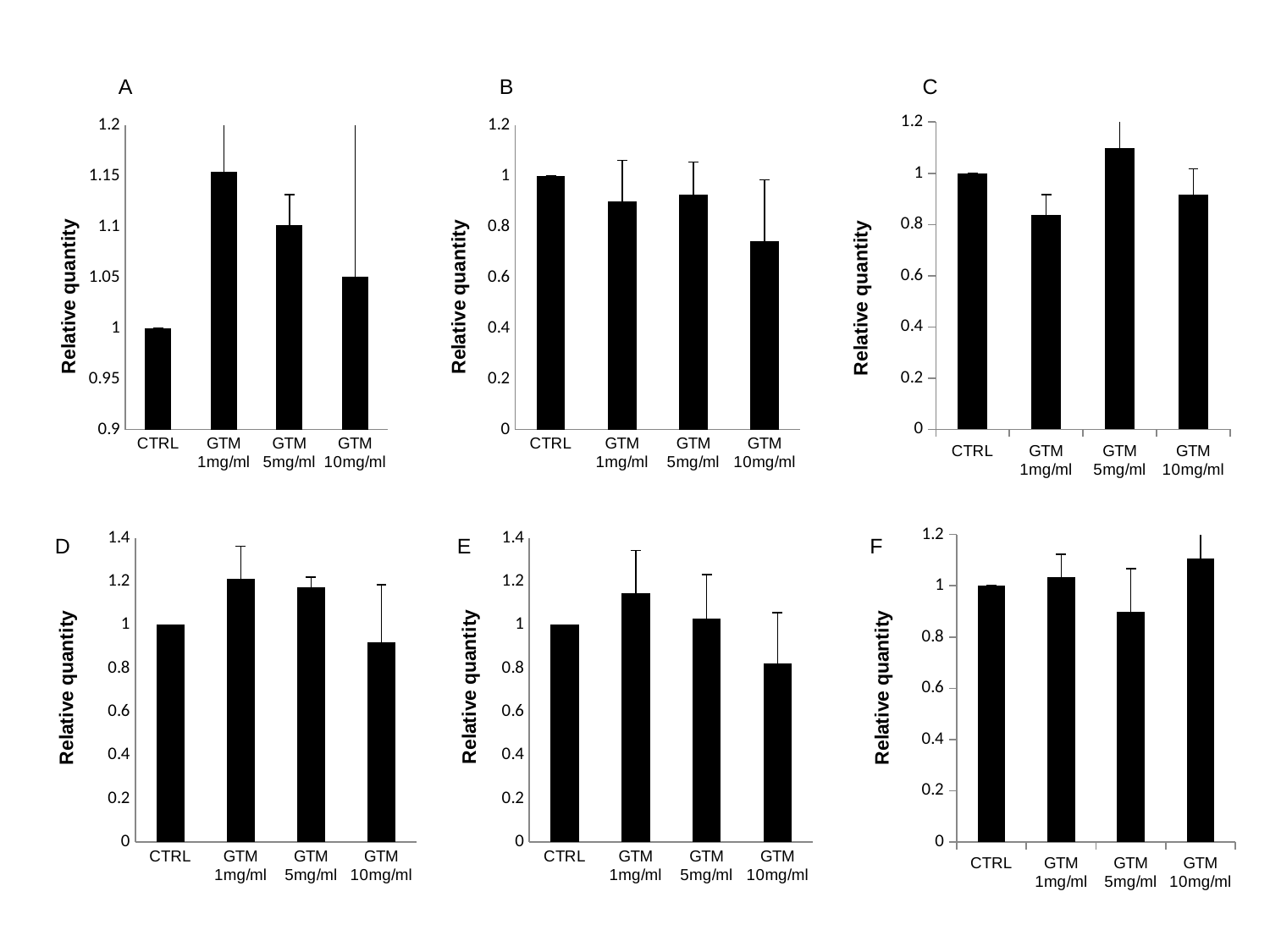

A
B
C
### Chart
| Category | |
|---|---|
| CTRL | 1.0 |
| GTM 1mg/ml | 1.154514996693852 |
| GTM 5mg/ml | 1.10168670104246 |
| GTM 10mg/ml | 1.050821697534136 |
### Chart
| Category | |
|---|---|
| CTRL | 1.0 |
| GTM 1mg/ml | 0.901488512001829 |
| GTM 5mg/ml | 0.925623626867157 |
| GTM 10mg/ml | 0.744136263915955 |
### Chart
| Category | |
|---|---|
| CTRL | 1.0 |
| GTM 1mg/ml | 0.836463080243672 |
| GTM 5mg/ml | 1.09946862825827 |
| GTM 10mg/ml | 0.916848656285433 |
### Chart
| Category | |
|---|---|
| CTRL | 1.0 |
| GTM 1mg/ml | 1.21284502506496 |
| GTM 5mg/ml | 1.173290781408283 |
| GTM 10mg/ml | 0.919145325540542 |
### Chart
| Category | |
|---|---|
| CTRL | 1.0 |
| GTM 1mg/ml | 1.146670155850274 |
| GTM 5mg/ml | 1.029818149272171 |
| GTM 10mg/ml | 0.820139512291055 |
### Chart
| Category | |
|---|---|
| CTRL | 1.0 |
| GTM 1mg/ml | 1.033031571928031 |
| GTM 5mg/ml | 0.89669798734631 |
| GTM 10mg/ml | 1.107554116766116 |
